# Biochemical and Structural Insights on the Poplar Tau Glutathione Transferase GSTU19 and 20 Paralogs Binding Flavonoids

**DOI:** 10.3389/fmolb.2022.958586

**Published:** 2022-08-12

**Authors:** Elodie Sylvestre-Gonon, Laura Morette, Morgane Viloria, Sandrine Mathiot, Alexis Boutilliat, Frédérique Favier, Nicolas Rouhier, Claude Didierjean, Arnaud Hecker

**Affiliations:** ^1^ Université de Lorraine, INRAE, IAM, Nancy, France; ^2^ Université de Lorraine, CNRS, CRM2, Nancy, France

**Keywords:** flavonoids, glutathione transferase (GST), poplar, *Populus trichocarpa*, structure, ligandin property, specialized metabolism, photosynthetic organisms

## Abstract

Glutathione transferases (GSTs) constitute a widespread superfamily of enzymes notably involved in xenobiotic detoxification and/or in specialized metabolism. *Populus trichocarpa* genome (V4.1 assembly, Phytozome 13) consists of 74 genes coding for full-length GSTs and ten likely pseudogenes. These GSTs are divided into 11 classes, in which the tau class (GSTU) is the most abundant with 54 isoforms. PtGSTU19 and 20, two paralogs sharing more than 91% sequence identity (95% of sequence similarity), would have diverged from a common ancestor of *P. trichocarpa* and *P. yatungensis* species. These enzymes display the distinctive glutathione (GSH)-conjugation and peroxidase activities against model substrates. The resolution of the crystal structures of these proteins revealed significant structural differences despite their high sequence identity. PtGSTU20 has a well-defined deep pocket in the active site whereas the bottom of this pocket is disordered in PtGSTU19. In a screen of potential ligands, we were able to identify an interaction with flavonoids. Some of them, previously identified in poplar (chrysin, galangin, and pinocembrin), inhibited GSH-conjugation activity of both enzymes with a more pronounced effect on PtGSTU20. The crystal structures of PtGSTU20 complexed with these molecules provide evidence for their potential involvement in flavonoid transport in *P. trichocarpa*.

## Introduction

Glutathione transferases (GSTs) constitute a widespread superfamily of versatile enzymes able to modify a broad range of molecules by catalyzing diverse enzymatic reactions. These include glutathione (GSH)-conjugation, peroxidase, thiol-transferase, deglutathionylation, and dehydroascorbate reductase activities (Garcerá et al., 2006; Federici et al., 2009; Lallement et al., 2016; Gonzalez et al., 2018). In addition to their catalytic properties, these enzymes transport molecules through noncatalytic ligandin properties. They are involved in the protection of cells from a wide range of biotic and abiotic stresses such as UV radiation or pathogen attacks by participating either in the detoxification of toxic compounds (often referred to as xenobiotics) or in the synthesis of secondary defense metabolites. At the structural level, most GSTs are dimers and each protomer consists of an N-terminal thioredoxin domain and a C-terminal all helical domain. The N-terminal and C-terminal domains contain most of the residues that participate in the binding of GSH (G-site) and the hydrophobic substrate (H-site), respectively. Both sites (G- and H-sites) constitute the active site of the enzyme. Depending on the conserved residue (usually serine or cysteine in plant isoforms) present in their catalytic site, GSTs have opposite activities. While serinyl-GSTs catalyze GSH-conjugation reaction, cysteinyl-GSTs catalyze the deglutathionylation of small molecules. In plants, GSTs are divided into at least 14 classes, namely, lambda (L), hemerythrin (H), iota (I), ure2p, glutathionyl-hydroquinone reductase (GHR), elongation factor 1B gamma (EF1Bγ), dehydroascorbate reductase (DHAR), metaxin (MTX), microsomal prostaglandin E synthase type-2 (mPGES-2), phi (F), tau (U), tetrachlorohydroquinone dehalogenase (TCHQD), theta (T), and zeta (Z) classes (Lallement et al., 2014). In the model tree *Populus trichocarpa* (poplar), more than 80 GST isoforms were identified and categorized into 11 classes, with ure2p, iota, and hemerythrin representatives being absent. While there are only two to three members in each GST class in plants, members of tau and phi classes are overrepresented and the corresponding genes are often organized in clusters. In *P. trichocarpa*, there are 54 GSTU and 8 GSTF isoforms. This expansion is the result of several duplication events that occurred during evolution (Jain et al., 2010; Lan et al., 2010; Lallement et al., 2014; Dong et al., 2016; He et al., 2016; Xu et al., 2017; Han et al., 2018; Islam et al., 2018; Khan et al., 2018). We recently proposed that these gene duplication events could either create functional redundancy between GST isoforms, making it difficult to study their biological functions using reverse-genetic approaches, or, on the contrary, generate structural and functional diversity upon accumulation of mutations on certain genes, less subject to evolutionary pressure (Sylvestre-Gonon et al., 2019). The idea of functional redundancy between orthologs is often considered for functional annotation of new released genome, but whether this assumption is true remains to be validated experimentally case by case. A recent study conducted on three poplar species including *P. euphratica*, *P. trichocarpa*, and *P. yatungensis* led to the identification of 21 GST orthologous groups (Yang et al., 2019). Although the sequences of these orthologous groups are well preserved, nonsynonymous substitutions at key amino acid sites play an important role in the divergence of enzyme functions. In order to validate that the homolog/paralog redundancy rule applies to members of the poplar GST family, we focus, in this study, on the two *P. trichocarpa* paralogous proteins PtGSTU19 and PtGSTU20. After production, in *Escherichia coli*, and purification of these proteins as recombinant proteins, their biochemical and structural properties were analyzed and compared. Despite the high conservation of their primary sequences and enzymatic signatures, we observed that PtGSTU19 and PtGSTU20 paralogs display substantial structural differences. In a screen for identifying potential ligands, we were able to identify interactions with flavonoids. These interactions were confirmed by inhibition tests and by crystallographic studies of PtGSTU20 in complex with flavonoids.

## Materials and Methods

### Cloning, Site-Directed Mutagenesis, Protein Expression, and Purification

Sequences coding for PtGSTU19 (Potri.008G174900) and PtGSTU20 (Potri.008G175000) were amplified by PCR from poplar cDNA using specific primers (Supplementary Table S1) and cloned into pET-12a plasmid (Novagen) between *Nde*I and *Bam*HI restriction sites. Site-directed mutagenesis was performed using mutagenic oligonucleotides (Supplementary Table S1) and the QuikChange site-directed mutagenesis kit (Agilent Technologies).

Intact and mutated proteins were expressed in *E. coli* BL21 (DE3) expression strain (Novagen) containing the pSBET plasmid (expressing the AGG- and AGA-recognizing tRNA) upon transformation with the recombinant pET-12a plasmids. Bacteria were grown at 37°C in LB medium supplemented with kanamycin (50 μg ml^−1^) and ampicillin (50 μg ml^−1^) until the cell culture reached an OD_600nm_ of 0.7–0.8. Recombinant protein expression was then induced with 0.1 mM isopropyl β-d-1-thiogalactopyranoside and cells were further grown for 4 h. Cells were harvested by centrifugation, resuspended in a 30 mM Tris-HCl pH 8.0, 1 mM EDTA, and 200 mM NaCl lysis buffer, and stored at −20°C. Cell lysis was completed by sonication and the cell extract further centrifuged at 40,000 g for 25 min at 4°C to remove cellular debris and aggregated proteins. The proteins contained in the supernatant were then precipitated with ammonium sulfate to successively 40% and 80% of the saturation. After SDS-PAGE analysis on a 15% gel under reducing conditions, the fraction containing the majority of the recombinant proteins was subjected to size-exclusion chromatography by loading the protein extract on an Ultrogel^®^ ACA44 (5 × 75 cm, Biosepra) column equilibrated with a 30 mM Tris-HCl pH 8.0, 1 mM EDTA, and 200 mM NaCl buffer. The fractions containing the recombinant proteins were pooled and dialyzed by ultrafiltration to remove salt and loaded onto a DEAE-cellulose column (Sigma Aldrich) equilibrated with a 30 mM Tris-HCl pH 8.0 and 1 mM EDTA buffer. Recombinant proteins were eluted using a 0–0.4 M NaCl gradient, dialyzed, and further concentrated. The protein purity was analyzed by 15% SDS-PAGE under reducing conditions and protein concentrations were determined after measuring the absorbance at 280 nm using a theoretical molar absorption coefficient of 46,410 M^−1^ cm^−1^ for PtGSTU19; 43,430 M^−1^ cm^−1^ for PtGSTU20; and 44,920 M^−1^ cm^−1^ for PtGSTU19Y160A, PtGSTU19Y160C, PtGSTU19Y160F, and PtGSTU20C160Y variants. Recombinant proteins were finally stored at −20°C in 30 mM Tris-HCl pH 8.0 and 200 mM NaCl buffer until use.

### Measurement of Enzymatic Activities

The GSH-conjugation activity was assayed toward phenethyl-isothiocyanate (PITC), benzyl-isothiocyanate (BITC), 1-chloro-2,4-dinitrobenzene (CDNB), 4-hydroxy-2-nonenal (HNE), and 4-nitrophenyl-butyrate (PNP-butyrate) at 25°C in a final volume of 500 µL as described previously (Pégeot et al., 2014). Various concentrations of PITC (5–500 μM), HNE (6.25–175 μM), CDNB (125–5000 μM), BITC (7.5–900 μM), and PNP-butyrate (50–2500 μM) were tested at a fixed, saturating GSH concentration of 1 mM. When using HNE as a substrate, the GSH concentration was fixed at 0.7 mM to limit interferences with HNE at 224 nm. Peroxidase activity toward hydroperoxides was measured using an NADPH coupled spectrophotometric assay (Pégeot et al., 2014). The reactions were carried out in 500 μL of 30 mM Tris-HCl pH 8.0, 1 mM EDTA containing 150 μM NADPH, 0.5 unit of yeast glutathione reductase, various concentrations of peroxides (12.5–2000 μM), and a fixed concentration of 1 mM of GSH.

Competition assays were performed toward PITC in the presence of flavonoids (baicalein, chrysin, galangin, morin, pinocembrin, and pinostrobin) as well as glutathionyl-phenylacetophenone (GS-PAP) in a final volume of 500 µL. When flavonoid solubility was compatible with the assay (baicalein, morin, pinocembrin, pinostrobin, or GS-PAP), tests were assayed in 100 mM pH 6.4 phosphate buffer containing various concentrations of PITC (5–400 µM), a fixed concentration of GSH (1 mM), and various concentrations of flavonoids ranging from 0 to 200 µM.

The measured velocities were corrected by subtracting the rate of the spontaneous nonenzymatic reaction, and three independent experiments were performed at each substrate concentration. The kinetic parameters (*k*_cat_ and apparent *K*_m_) and the inhibition constants (*K*_i_) were, respectively, obtained by fitting the data to the nonlinear regression Michaelis–Menten model (kinetic assays) and to the mixed inhibition model (inhibition assays) in GraphPad Prism 8 software (Copeland, 2013). The *k*_cat_ values were expressed as μmol of substrate oxidized per second per μmol of enzyme (*i.e*., the turnover number in s^−1^), using specific molar absorption coefficients of 9600 M^−1^ cm^−1^ at 340 nm for CDNB, 9250 M^−1^ cm^−1^ at 274 nm for BITC, 8890 M^−1^ cm^−1^ at 274 nm for PITC, 17,700 M^−1^ cm^−1^ at 412 nm for PNP-butyrate, 13,750 M^−1^ cm^−1^ at 224 nm for HNE, and 6220 M^−1^ cm^−1^ at 340 nm for NADPH.

### Identification of Potential PtGSTU19 and 20 Ligands by Thermal Shift Assays

The experiments were performed in 96-well microplates (Harshell, Biorad) and the measurements carried out using a real-time PCR detection system (CFX 96 touch, Biorad) (Cimmperman and Matulis, 2011). Assays were performed in a mixture (final volume of 25 µL) containing 30 mM Tris-HCl pH 8.0, 100 µM of chemical compounds (diluted in 8% DMSO) originating from a chemical library (Supplementary Tables S2, S3), 20 µM of PtGSTU19 or 20, and 5X SYPRO orange. Fluorescence was measured each minute at 530 nm after excitation at 485 nm starting after 3-min incubation at 5°C and increasing the temperature from 5 to 95°C with steps of 1°C per minute. The denaturation temperature, which corresponds to the temperature at which 50% of the total fluorescence is measured, was determined by the nonlinear regression Boltzmann sigmoidal model in GraphPad Prism 8 software for data obtained in the presence of potential ligands. This temperature was compared with a reference assay in which organic compounds were replaced by an equivalent DMSO concentration.

### Crystallization and Structural Determination of Recombinant PtGSTU19 and 20

The pre-crystallization test (PCT from Hampton Ltd.) was used to determine the most promising range of protein concentrations for the initial screenings (10–20 mg/ml for PtGSTU19 and 20–40 mg/ml for PtGSTU20). Preliminary crystallization conditions were found with Oryx 8 robot (Douglas Instruments Ltd.) of the CRM2 crystallogenesis platform (University of Lorraine). The screens were performed in 96-well plates using the sitting-drop vapor-diffusion method (Chayen, 1998) with purchased crystallization kits (Wizard™ Classic kits 1–4 from Rigaku Ltd., Structure Screens 1–2 from Molecular Dimension Ltd., and Classic kits 1–10 and JCSG kit from JENA Bioscience Ltd., 624 conditions). Both protein solutions contained 30 mM Tris-HCl pH 8.0 and 1 mM EDTA. Crystallization plates were stored at 4°C. Three and four conditions yielded crystals for PtGSTU19 (Sts 1–15, Sts 1–20, and JSB 2-C4) and PtGSTU20 (JBS 2-B6, JBS 2-C4, JBS 2-D5, and PCT B2), respectively. The crystals were optimized manually using the microbatch under oil method (Chayen, 1998) with the conditions Sts 1–15 and Sts 1–20 for PtGSTU19 and with the condition JBS 2-B6 for PtGSTU20. Suitable crystals for X-ray diffraction were obtained by varying the protein/condition volume ratio (1 μL/2 μL, 1.5 μL/1.5 μL, and 2 μL/1 μL). Sts 1–15 condition contains 0.2 M magnesium acetate tetrahydrate, 0.1 M sodium cacodylate pH 6.5, and 20% w/v PEG 8000. Sts 1–20 condition contains 0.2 M calcium acetate hydrate, 0.1 M sodium cacodylate pH 6.5, and 18% w/v PEG 8000. JBS 2-B6 condition contains 200 mM calcium chloride, 100 mM Tris-HCl pH 8.5, and 20% w/v PEG 4000. Crystals of PtGSTU19/20-GSH (U19^GSH^ and U20^GSH^), PtGSTU20-glutathionyl-phenylacetophenone (U20^GS-PAP^), and PtGSTU20-flavonoids (U20-galangin U20^GAL^, U20-morin U20^MOR^, U20-baicalein U20^BAI^, and U20-pinocembrin U20^PIN^) were obtained by co-crystallization using a ligand concentration of 5 mM. All crystals were flash frozen in a liquid nitrogen stream at 100 K after a quick soaking in their mother liquor supplemented with 20% glycerol.

Preliminary X-ray diffraction experiments were carried out in-house on an Agilent SuperNova diffractometer (Oxford Diffraction) equipped with a CCD detector and data further collected at SOLEIL synchrotron on beamlines PROXIMA-1 and -2 (Gif Sur Yvette, France) or at ESRF synchrotron on beamline ID30a-3 (Grenoble, France). Data sets were indexed and integrated with XDS (Kabsch, 2010), scaled, and merged with Aimless (Evans and Murshudov, 2013) from the CCP4 suite (Winn et al., 2011). The structure of PtGSTU19 was solved by molecular replacement using MOLREP (Vagin and Teplyakov, 2010) with the coordinates of GSTU from *Ricinus communis* (PDB code 4J2F) as the search model. The structure of PtGSTU20 was solved by molecular replacement using MOLREP with the coordinates of PtGSTU19 as the search model. For all complexes, difference Fourier maps revealed unambiguously the presence of the ligands in the active site of the protein. Structures were then refined with Buster (Smart et al., 2012) and manually adjusted with Coot (Emsley and Cowtan, 2004). Validation of all structures was performed with MolProbity (Davis et al., 2004) and the wwPDB validation server (http://validate.wwpdb.org). Crystal data, diffraction, and refinement statistics are shown in Table 1 and all structural figures were generated with Pymol (Schrödinger LLC). Coordinates and structural factors have been deposited in the Protein Data Bank (PDB ID: 7ZS3 (U19^APO^), 7ZVP (U19^GSH^), 7ZZN (U20^APO^), 8A08 (U20^GSH^), 8A0I (U20^GS-PAP^), 8A0Q (U20^BAI^), 8A0O (U20^GAL^), 8A0P (U20^MOR^), and 8A0R (U20^PIN^)).

**TABLE 1:** Statistics of X-ray diffraction data collection and model refinement.

	PtGSTU19		PtGSTU20						
						Flavonols		Flavones	Flavanones
	**Apo**	**GSH**	**Apo**	**GSH**	**GS-PAP**	**Galangin**	**Morin**	**Baicalein**	**Pinocembrin**
	**U19** ^**APO**^	**U19** ^**GSH**^	**U20** ^**APO**^	**U20** ^**GSH**^	**U20** ^**GS-PAP**^	**U20** ^**GAL**^	**U20** ^**MOR**^	**U20** ^**BAI**^	**U20** ^**PIN**^
**Data collection**									
Diffraction source	SOLEIL-Px1	SOLEIL-Px2	SOLEIL-Px2	SOLEIL-Px2	SOLEIL-Px2	ESRF-ID30a3	SOLEIL-Px2	SOLEIL-Px1	ESRF-ID30a3
Detector	EIGER X 16M	EIGER X 9M	EIGER X 9M	EIGER X 9M	EIGER X 9M	Eiger X 4M	EIGER X 9M	EIGER X 16M	Eiger X 4M
Wavelength (Å)	0.97857	0.98010	0.980106	0.98011	0.98012	0.967700	0.98012	0.97856	0.967700
Space group	*P*4_1_2_1_2	*P*4_1_2_1_2	*P*4_1_2_1_2	*P*4_1_2_1_2	*P*4_1_2_1_2	*P*4_1_2_1_2	*P*4_1_2_1_2	*P*4_1_2_1_2	*P*4_1_2_1_2
Unit-cell a; c (Å)	56.8; 182.3	56.7; 182.0	56.0; 183.2	56.6; 181.6	56.6; 181.8	56.6; 181.0	56.6; 183.0	56.1; 181.6	56.3; 182.6
Resolution range (Å)	41.5–1.6 (1.63–1.60)	48.1–1.6 (1.64–1.61)	47.8–2.0 (2.06–2.01)	48.0–1.6 (1.67–1.64)	45.5–1.6 (1.67–1.62)	41.3–1.8 (1.88–1.84)	48.1–1.7 (1.73–1.69)	47.7–2.1 (2.05–2.10)	50.0–1.6 (1.69–1.60)
Tot. no. of meas. int	719,996 (26,911)	381,726 (19,190)	517,173 (34,020)	945,416 (39,869)	976,401 (64,399)	691,036 (41,072)	879,097 (57,188)	493,731 (34,941)	1,068,173 (152,671)
Unique reflections	40,239 (1870)	39,646 (1927)	20,354 (1413)	36,971 (1740)	38,378 (2678)	26,724 (1560)	34,640 (2397)	19,240 (1370)	40,026 (5669)
Average redundancy	18 (14)	10 (10)	25 (25)	26 (23)	25 (24)	26 (26)	25 (24)	26 (26)	27 (28)
Mean *I*/σ(*I*)	22.5 (2.1)	17.7 (2.3)	31.8 (2.2)	33.3 (2.4)	28.6 (2.1)	19.4 (3.3)	34.5 (2.2)	35.2 (4.0)	28.5 (3.0)
Completeness (%)	99.7 (95.2)	99.9 (100)	99.7 (96.1)	99.8 (96.1)	99.7 (95.5)	99.8 (97.5)	99.7 (95.9)	99.9 (98.2)	100.0 (100.0)
*R*_merge_	0.068 (1.40)	0.062 (0.67)	0.057 (1.674)	0.055 (1.44)	0.064 (1.308)	0.102 (1.101)	0.049 (1.380)	0.053 (0.80)	0.062 (1.118)
*R*_meas_	0.070 (1.46)	0.066 (0.70)	0.058 (1.709)	0.056 (1.47)	0.066 (1.339)	0.104 (1.122)	0.051 (1.410)	0.054 (0.81)	0.063 (1.139)
CC_1/2_	1.00 (0.80)	1.00 (0.94)	1.00 (0.89)	1.00 (0.83)	1.00 (0.78)	1.00 (0.85)	1.00 (0.80)	1.00 (0.96)	1.00 (0.89)
Wilson *B*-factor (Å^2^)	30.8	26.7	49.4	30.2	31.5	33.2	36.0	44.9	28.5
**Refinement**									
Resolution range (Å)	16.1 1.6	48.1 1.6	20.7 2.0	22.7 1.6	45.4 1.6	21.7 1.8	22.9 1.7	47.7 2.1	16.0 1.6
No. of reflections	40,092	35,549	20,247	36,853	38,281	26,615	34,524	19,138	39,871
*R*_work_/*R*_free_	0.227/0.235	0.229/0.250	0.228/0.253	0.204/0.235	0.210/0.232	0.215/0.231	0.215/0.231	0.226/0.256	0.214/0.231
Corr Fo–Fc/Fo–Fc_free_	0.949/0.951	0.948/0.942	0.939/0.938	0.952/0.939	0.953/0.954	0.944/0.940	0.951/0.950	0.937/0.914	0.951/0.945
Total number of atoms	2117	2070	1720	2091	2092	1942	1981	1801	2023
Average *B*-factor (Å^2^)	35.7	32.1	56.0	34.3	33.7	42.0	40.0	51.0	33.0
**Model quality**									
RMSZ bond lengths	0.43	0.43	0.42	0.42	0.42	0.42	0.42	0.42	0.42
RMSZ bond angles	0.53	0.53	0.55	0.56	0.54	0.50	0.53	0.52	0.54
Ramachandran fav. (%)	97	98	96	96	96	97	96	96	97
Ramachandran all. (%)	3	2	4	4	4	3	4	3	3
Rotamer outliers (%)	0	0	0	0	0	0	1	1	1
Clashscore	1	2	2	1	2	1	1	1	1
**PDB entry**	**7ZS3**	**7ZVP**	**7ZZN**	**8A08**	**8A0I**	**8A0O**	**8A0P**	**8A0Q**	**8A0R**

*R*_merge_ = $\underset{hkl}{\sum }\underset{i}{\sum }\left|I_{i}\left(hkl\right)-\;I\left(hkl\right)\right|/\underset{hkl}{\sum }\underset{i}{\sum }I_{i}\left(hkl\right)$ . *R*_meas_ = $\underset{hkl}{\sum }{\left\{N\left(hkl\right)/\left[N\left(hkl\right)-1\right]\right\}}^{1/2}\;\underset{i}{\sum }\left|I_{i}\left(hkl\right)-I\left(hkl\right)\right|/\underset{hkl}{\sum }\underset{i}{\sum }I_{i}\left(hkl\right)$ . CC_1/2_ is the correlation coefficient of the mean intensities between two random half-sets of data. *R*_work_ = $\underset{hkl}{\sum }\left|\left|F_{obs}\right|-\left|F_{calc}\right|\right|/\underset{hkl}{\sum }\left|F_{obs}\right|$ ; 5% of the reflections were selected for *R*_free_ calculation. RMSZ: root mean square *Z*-score. The MolProbity clashscore is the number of serious clashes per 1000 atoms. Values in parentheses are for the highest resolution shell.

## Results and Discussion

### Diversity of GSTUs From *Populus trichocarpa*

In a previous study, Lan et al. identified 81 putative genes coding for full-length GSTs in version 1.1 of *P. trichocarpa* genome (Lan et al., 2009). The tau and phi GSTs were the most represented with 58 and 9 members, respectively. Among these genes, 66 were located on 15 out of 19 chromosomes, while the other 15 genes were assigned to 14 scaffold fragments. The distribution of *GST* genes between chromosomes was uneven since chromosomes 7, 9, 17, and 18 harbor no *GST* gene unlike chromosomes 1, 8, 10, 11, 14, and 19 where *GST* genes were arranged in clusters. Among GSTU-encoding genes, 37 were arranged in six clusters (clusters I to V and VII) on chromosomes 1, 8, 10, 11, and 19 whereas four *GSTF* genes were in one cluster (cluster VI) on chromosome 14. Members of the minor GST classes were sparsely distributed in a single locus on different chromosomes. A reexamination of *P. trichocarpa* genome using the last annotated version (V4.1 assembly, Phytozome 13) prompted us to identify 74 genes coding for full-length GSTs categorized into 11 classes and 10 likely pseudogenes distributed on 17 out of 19 chromosomes. Chromosomes 7 and 9 do not contain any gene coding for GSTs (Supplementary Figure S1). Tau and phi GSTs are the most represented and are encoded by 44 and 8 genes, respectively. Among them, 39 *GSTU* genes and 4 *GSTF* genes are organized into 5 main clusters distributed on chromosomes 1, 8, 10, 11 (*GSTU* clusters), and 2 (*GSTF* clusters). At the sequence level, the 43 full-length poplar GSTUs present a conserved serine usually present at position 10 mostly included in an SPFX (X being a small aliphatic residue like alanine, valine, or glycine) or SP[F/Y][S/C] conserved signature (Supplementary Figure S2). Among the isoforms identified, one (Potri.011G140600) exhibits an atypical signature for which the conserved serine, which normally contributes to the lowering of the p*K*a of the thiol group of the GSH, is substituted by an alanine. The absence of this conserved serine may be compensated by the presence of two adjacent serinyl residues in positions 3 and 4 of the catalytic motif APSS. Such a situation was already described for some poplar GSTFs (Pégeot et al., 2017). None of the GSTU sequences identified in poplar possesses a recognizable targeting sequence, suggesting that all these proteins are likely cytosolic as already suggested for other GSTU proteins (Dixon et al., 2009). Among poplar *GSTU* genes, Potri.008G174900 and Potri.008G175000, which, respectively, code for PtGSTU19 and PtGSTU20, are two adjacent and paralogous genes that share 91% sequence identity (Supplementary Figures S1, S2). Yang *et al.* (2019) recently studied *GST* genes in three closely related *Populus* species: *P. trichocarpa*, *P. yatungensis*, and *P. euphratica*. *P. trichocarpa* is closer to *P. yatungensis* than to *P. euphratica*, and the lineages of *P. trichocarpa* and *P. euphratica* would have diverged *c.* 8–11 Ma ago (Ma et al., 2013; Yang et al., 2019). Yang *et al.* (2019) showed that PtGSTU19 and U20 isoforms result from a duplication event of a common ancestor of the three species and that a more important divergence has occurred more recently in a common ancestor of *P. trichocarpa* and *P. yatungensis*. Indeed, *P. yatungensis* has two paralogs (PyGSTU19 and PyGSTU20) that show 91% sequence identity (95% of sequence similarity) as observed in *P. trichocarpa*. *P. euphratica* also has two, but the identity is higher (97%, six residues differ), and the two paralogs (PeGSTU19 and PeGSTU20) are PtGSTU19-like (93% and 90% sequence identities with PtGSTU19 and 20, respectively).

### Both PtGSTU19 and 20 Present a Typical Enzymatic Signature of Ser-GSTs

To get insight into the biochemical and structural properties of both PtGSTU19 and PtGSTU20, we produced the corresponding untagged recombinant proteins using a bacterial heterologous system (*E. coli*) and purified them by a three-step purification strategy combining ammonium sulfate precipitation, exclusion chromatography, and ion-exchange chromatography. Around 50 mg of soluble protein per liter of culture was obtained for both proteins (Supplementary Figure S3A). Molecular masses of 24,892 and 24,796 Da, which are compatible with the theoretical molecular masses after removal of the start methionine (131 Da), were obtained by mass spectrometry for the purified PtGSTU19 and PtGSTU20 recombinant proteins, respectively. SEC-MALS analysis revealed that they adopt a dimeric arrangement as expected for typical GSTUs (Supplementary Figure S3B) (Pégeot et al., 2014, 2017). Retention tests on GSH-Sepharose affinity chromatography showed that, in contrast to PtGSTU20, recombinant PtGSTU19 was partially retained by the resin indicating that its GSH-binding site (G-site) is partially occupied by a molecule that is most likely GSH (Supplementary Figure S3C).

As PtGSTU19 and 20 possess a conserved serine in their active site, we then explored their enzymatic properties using typical Ser-GST substrates. GSH-conjugation activity was assayed toward CDNB, PITC and BITC, PNP-butyrate, and HNE (Table 2). A GSH-conjugating activity was detected for both PtGSTU19 and PtGSTU20 toward all substrates tested. Catalytic efficiencies of PtGSTU19 ranged from 2.0 × 10^3^ M^−1^ s^−1^ for HNE to 132.2 × 10^3^ M^−1^ s^−1^ for BITC and those of PtGSTU20 from 0.3 × 10^3^ M^−1^ s^−1^ for CDNB to 88.9 × 10^3^ M^−1^ s^−1^ for PITC. The more marked activity of both enzymes on isothiocyanates is due to a better apparent affinity (*K*_m_) of the enzymes for substrates and a pretty good turnover (*k*_cat_) number of the enzyme for PITC and BITC. On the contrary, the catalytic activity measured with PNP-butyrate is quite low for both enzymes (100 M^−1^ s^−1^) due to a lower affinity for the substrate and a lower turnover number. Such observation has been documented with the poplar GSTU16 (*P. trichocarpa*) (Musdal and Mannervik, 2015) and with *Arabidopsis thaliana* GSTU4, 6, 10, 12, and 13 (Dixon et al., 2009). The sole substantial catalytic difference observed between PtGSTU19 and 20 was toward CDNB for which the catalytic efficiency of PtGSTU19 was 30 times higher than the one of PtGSTU20 but it remains of the same order of magnitude as those obtained for GSTU2 and GSTU9 from *Larix kaempferi* (Yang et al., 2014).

**TABLE 2:** Kinetic parameters of PtGSTU19 and 20 toward model substrates.

	CDNB	PITC	BITC	PNP-butyrate	HNE	CuOOH
*k*_cat_ (s^-1^)						
PtGSTU19	28.8 ± 2.2	3.8 ± 0.1	7.0 ± 0.1	0.040 ± 0.001	0.11 ± 0.01	0.17 ± 0.01
PtGSTU20	0.59 ± 0.03	5.1 ± 0.1	10.4 ± 0.3	0.020 ± 0.001	0.11 ± 0.01	0.24 ± 0.01
*K*_m_ (µM)						
PtGSTU19	3394 ± 502	48.4 ± 2.5	52.7 ± 3.6	443.5 ± 31.1	56.4 ± 7.4	196.5 ± 31.1
PtGSTU20	1777 ± 193	56.8 ± 4.1	149.6 ± 17.6	329.2 ± 28.1	30.6 ± 3.5	98.6 ± 15.4
*k*_cat_/*K*_m_ (10^3^ M^-1^s^-1^)						
PtGSTU19	8.9 ± 0.8	79.0 ± 1.1	132.2 ± 0.2	0.100 ± 0.002	2.0 ± 0.1	0.85 ± 0.04
PtGSTU20	0.33 ± 0.01	88.9 ± 1.8	69.8 ± 2.2	0.060 ± 0.003	3.6 ± 0.1	2.4 ± 0.1

The apparent *K*_m_ values for all compounds were determined for PtGSTU19 and 20 by the varying substrate concentrations at a fixed saturating GSH concentration. The apparent *K*_m_ and *k*_cat_ values were calculated with GraphPad Prism 8 software using the Michaelis–Menten equation as nonlinear regression model. Results are means ± S.D. (*n* = 3).

Peroxidase activity was also assayed using cumene hydroperoxide (CuOOH), tert-butyl hydroperoxide (*t*-BOOH), and hydrogen peroxide (H_2_O_2_), as Ser-GSTs often reduce peroxides (Kilili et al., 2004; Axarli et al., 2009b; Pégeot et al., 2017). PtGSTU19 and 20 were weakly active on CuOOH (Table 2) but not toward *t*-BOOH and H_2_O_2_ even after increasing the enzyme concentration up to 10 µM. Catalytic efficiencies toward CuOOH remain low with 800 M^−1^ s^−1^ and 2400 M^−1^ s^−1^ for PtGSTU19 and 20, respectively.

Overall, we can notice that the enzymatic behaviors of both enzymes are quite similar and are comparable to other characterized Ser-GSTs from plants (Cummins et al., 2003; Axarli et al., 2009a; Lo Piero et al., 2010; Chronopoulou et al., 2012, 2014; Liu et al., 2013; Pégeot et al., 2017; Valenzuela-Chavira et al., 2017), insects, and fungi, as well as mammals (Sawicki et al., 2003; Wang et al., 2011; Mathieu et al., 2013; Gonzalez et al., 2018; Hu et al., 2020) whose activities range from 10^2^ to 10^7^ M^−1^ s^−1^ for GSH-conjugation activity and from 10^2^ to 10^3^ M^−1^ s^−1^ for peroxidase activity.

### Structural Analysis of Both PtGSTU19 and 20 Reveals Structural Differences

Crystallographic analysis of both PtGSTU19 and PtGSTU20 was initiated to get further insights into the structure–function relationships of these two paralogs. The crystal structures of PtGSTU19 and 20 were solved in their apo form (U19^**APO**^ and U20^**APO**^) and in complex with GSH (U19^**GSH**^ and U20^**GSH**^). In addition, we solved the crystal structure of PtGSTU20 in complex with glutathionyl-phenylacetophenone (U20^GS-PAP^). All the crystals of PtGSTU19 and 20 were isomorphic. The space group is *P*4_1_2_1_2 and the asymmetric unit contains one monomer (Table 1). The dimer axes coincide with the crystallographic dyads.

**FIGURE 1:**
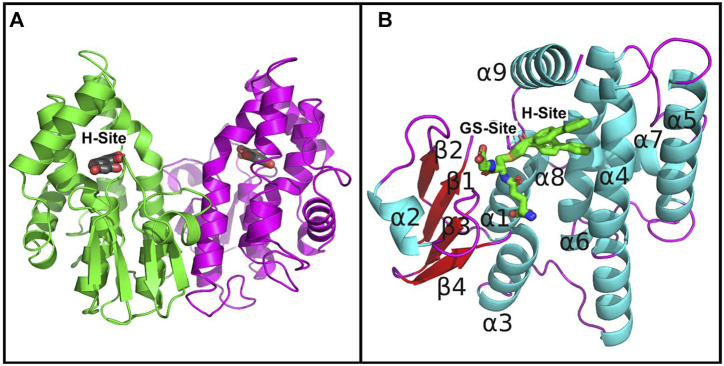
Crystal structures of PtGSTU20. **(A)** View of the dimer of PtGSTU20 that highlights its putative H-site. The backbone atoms (cartoon) are colored by monomer. The morin flavonoid is represented as gray sticks with the non-carbon atoms colored according to their types (red, oxygen; blue, nitrogen; yellow, sulfur). **(B)** View of PtGSTU20 monomer that shows both G- and H-sites. The G-site is occupied by the glutathionyl moiety of glutathionyl-phenylacetophenone (GS-PAP). The phenylacetophenone moiety, disordered over two positions, defines the boundaries of the putative H-site. The backbone atoms (cartoon) of PtGSTU20 are colored according to their secondary structure (cyan, helix; red, strand; magenta, loop). GS-PAP is represented as gray sticks with the non-carbon atoms colored according to their types (red, oxygen; blue, nitrogen; yellow, sulfur).

**FIGURE 2:**
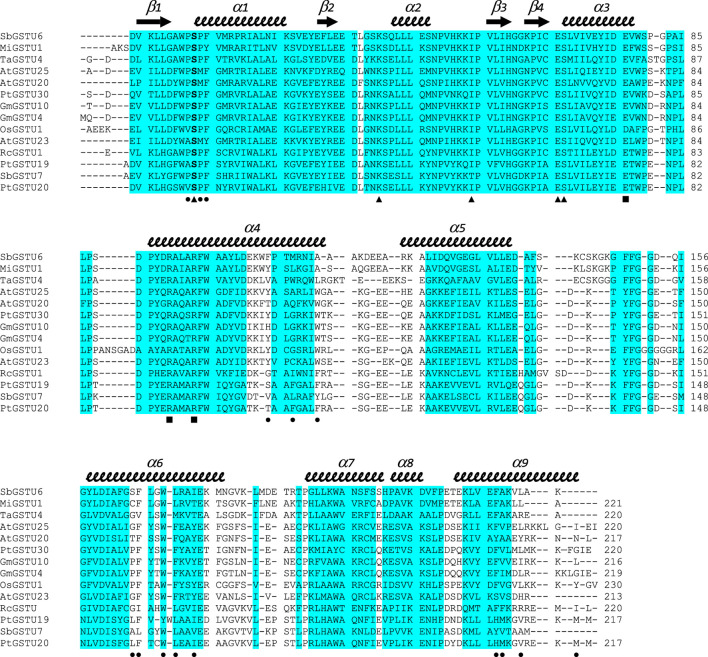
Structure-based sequence alignment of GSTUs highlighting common regions. The sequence alignment was generated with mTM-align. Sequences were retrieved from the RCSB PDB: SbGSTU6 (GSTU6 from *Salix babylonica*, PDB ID 7DW2), MiGSTU1 (GSTU1 from *Mangifera indica*, 5G5E), TaGSTU4 (GSTU4 from *Aegilops tauschii*, 1GWC), AtGSTU25 (GSTU25 from *Arabidopsis thaliana*, 5G5A), AtGSTU20 (GSTU20 from *A. thaliana*, 5ECS), PtGSTU30 (GSTU30 from *P. trichocarpa*, 5J4U), GmGSTU10 (GSTU10 from *Glycine max*, 4CHS), GmGSTU4 (GSTU4 from *G. max*, 2VO4), OsGSTU1 (GSTU1 from *Oryza sativa*, 1OYJ), AtGSTU23 (GSTU23 from *A. thaliana*, 6EP7), RcGSTU1 (GSTU1 from *Ricinus communis*, 4J2F), PtGSTU19 (this study), SbGSTU7 (GSTU7 from *S. babylonica*, PDB ID 7DWD), and PtGSTU20 (this study). Secondary structures are labeled and shown using arrows (β-strands) and squiggles (helices). Common regions, *i.e.*, regions with no gap and with pairwise residue distances less than 4Å are highlighted in blue. Residues participating in dimer stabilization via polar interactions are marked with ■. Residues involved in glutathione binding (G-site) are marked with ▲. Residues involved in the putative H-site of PtGSTU20 are marked with ●.

Since PtGSTU19 and 20 share a high sequence identity (91%), their overall three-dimensional structures are very close as expected (RMSD of 0.36 Å between U19^**GSH**^ and U20^**GSH**^ monomers) (Supplementary Figure S4). However, PtGSTU19 and 20 structures have distinct structural properties (see later). The dimers adopt the usual open V-shaped structure of tau and omega GSTs (Figure 1) (Sylvestre-Gonon et al., 2019). The buried area at the interface is around 2300 Å^2^ and the polar interactions at the interface involve Glu_75_ with Arg_90_ and Arg_94_ in both structures. PtGSTU19 and U20 have the same secondary structures as the known GSTU structures and show the canonical cytosolic GST fold (Sylvestre-Gonon et al., 2019). Indeed, PtGSTU19 and 20 monomers contain an N-terminal thioredoxin domain (β1α1β2α2β3β4α3) followed by a bundle of six α-helices (α4α5α6α7α8α9) (Figure 1). In U19^GSH^ and U20^GSH^, a glutathione molecule is bound to the G-site and the binding residues are mainly carried by the N-terminal domain. The five residues involved in polar interactions with GSH (Ser_12_, Lys_39_, Ile_53_, Glu_65_, and Ser_66_) are quasi-invariant in the known GSTU structures (Figure 2 and Supplementary Figure S5). The side chain of the cysteine moiety of GSH exists as two rotamers in U20^GSH^ (Supplementary Figure S4). In U19^GSH^, GSH was found oxidized to sulfenic acid GSOH (Supplementary Figure S4). GSOH was likely formed during crystallization after oxidation of the thiol group of GSH as documented for the GSTU10 from *Glycine max* (Skopelitou et al., 2015). Indeed, a mass spectrometry analysis of the GSH sample used for co-crystallization confirmed the absence of GSOH traces. GSOH is naturally formed as an intermediate by the reaction of GSH with hydroperoxides. The pH was the main difference between the crystallization conditions of PtGSTU19 (pH 6.5) and 20 (pH 8.5). This difference of two units could explain the difference in the oxidation state of glutathione in the crystal structures (GSOH in PtGSTU19 and GSH in PtGSTU20).

**FIGURE 3:**
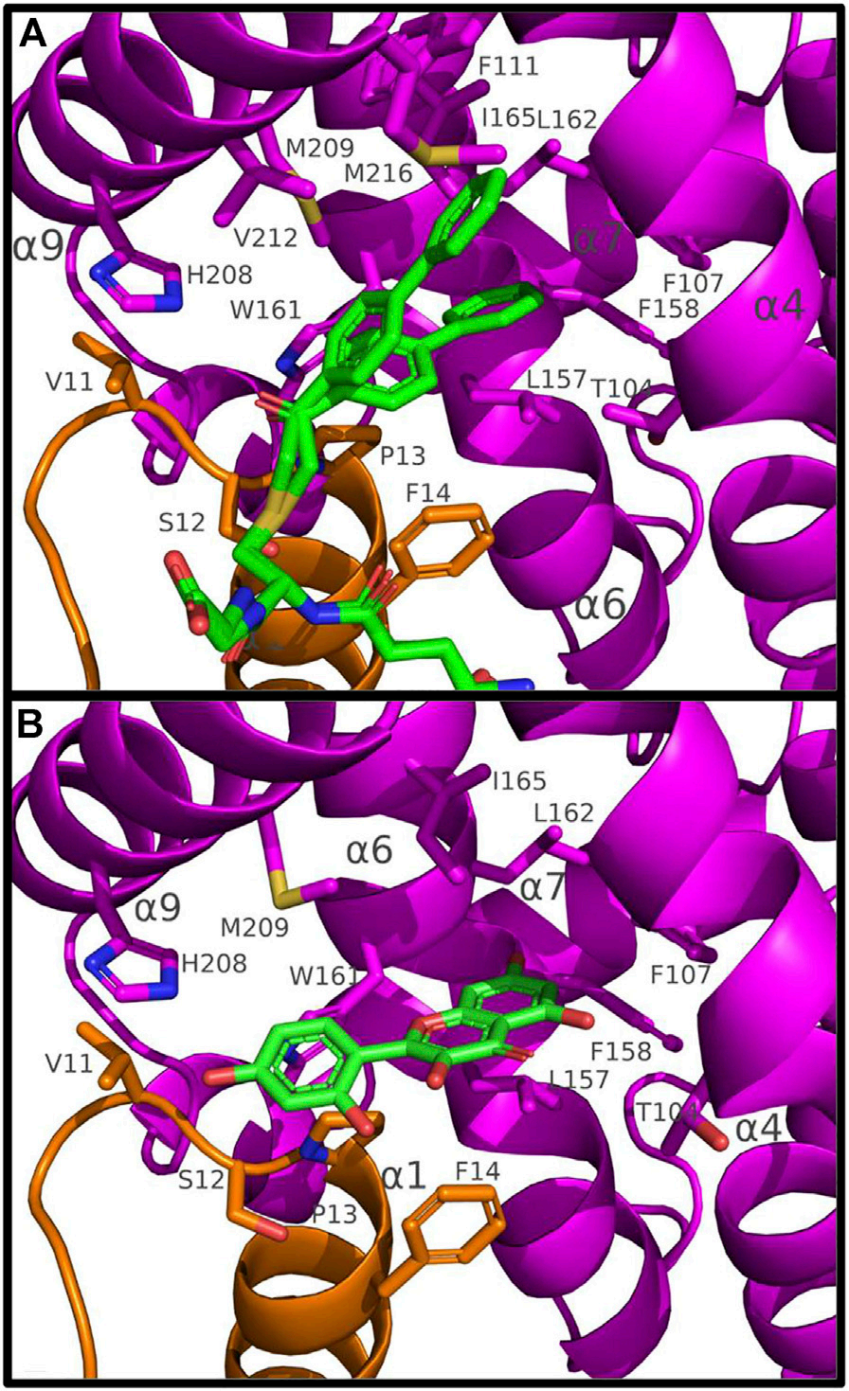
Binding of GS-PAP **(A)** and morin **(B)** in the putative H-site of PtGSTU20. Putative H-site of PtGSTU20 is a well-delineated cavity deeply inserted between the α4 and α6 helices of the C-terminal domain. Both conformations of GS-PAP are shown. The GS-PAP **(A)** and morin **(B)** ligands are represented in sticks as their surrounding residues. Intermolecular contacts are materialized as dashed lines. The N- and C-terminal domains are colored orange and magenta, respectively, and ligands are colored green. Non-carbon atoms are colored according to their types (red, oxygen; blue, nitrogen; yellow, sulfur).

In U20^GS-PAP^ complex, the glutathionyl moiety of the GS-PAP inhibitor is bound as glutathione in U19^GSH^ and U20^GSH^. The phenylacetophenone moiety adopts two conformations that delineate the putative H-site of PtGSTU20 (Figure 3). The pocket includes residues from the β1-α1 loop and the α1, α4, α6, and α9 helices (Figure 2). α6 helix has a conserved tryptophan residue in GSTUs (Trp_161_ in PtGSTU20) that is assumed to be one of the walls of the H-site (Figure 3) (Sylvestre-Gonon et al., 2019). In U20^APO/GSH/GS-PAP^, this tryptophan residue is buried (accessible surface area (ASA) of 4 Å^2^), making the H-site quite deep (Supplementary Figure S6). In a previous study on an omega GST from the saprophytic fungus *Trametes versicolor*, we showed that a deep H-site could be correlated with the ability of the GST protein to bind polyphenols like flavonoids (Schwartz et al., 2018). The H-site of PtGSTU19 has distinct properties. Nearly two-thirds of the helix α6 of U19^APO^ (residues Val_159_ to Gly_170_) were refined in two conformations (Supplementary Figure S6). The first conformation corresponds to the one found in U20^APO/GSH/GS-PAP^ with Trp_161_ buried (ASA of 10 Å^2^) and a deep H-site. In the second conformation, Trp_161_ is more exposed to the solvent (ASA of 44 Å^2^), which reduces the depth of the pocket (Supplementary Figure S6). In addition, the side chain of Tyr_160_ points in the direction of the assumed catalytic serine residue (Ser_12_). Only the second conformation persists in U19^GSH^. There is no strong polar interaction between the GSH and Tyr_160_ since the distance between Tyr_160_ OH atom and GSH SG atom is 6 Å. In this second conformation, the side chain of Tyr_160_ provides hydrophily to the H-site of PtGSTU19, which could have promoted glutathione oxidation (Supplementary Figure S7). A cysteine residue replaces Tyr_160_ in PtGSTU20 (Cys_160_) and only the first conformation is present whatever the form (U20^APO/GSH/GSP^). Site-directed mutagenesis was used to examine the catalytic importance of the residue at position 160, *i.e.*, at the bottom of the putative H-site of PtGSTU19 and U20. Substitution of PtGSTU19 Tyr_160_ by an alanine, cysteine, or phenylalanine residue (PtGSTU19Y160A, PtGSTU19Y160C, and PtGSTU19Y160F variants, respectively) and PtGSTU20 Cys_160_ by a tyrosine residue (PtGSTU20C160Y) did not affect (or slightly affected) the affinity for the electrophilic substrate and the turnover number of both enzymes (Supplementary Table S4). All recorded catalytic efficiencies and turnover numbers for these protein variants are in the same range as those determined for nonmutated enzymes. Only one significant difference was observed, which was difficult to rationalize. In PtGSTU19Y160F variant, the apparent affinity for glutathione was reduced by a factor 4 with PITC as electrophilic substrate while no variation was detected with BITC.

A comparison of structures with and without GSH revealed another difference between PtGSTU19 and PtGSTU20. In PtGSTU20^APO^, no signal was found in the electronic density map corresponding to the region between β2 and β3, which includes helix α2 and its upstream and downstream loops (residues Glu_34_ to Lys_51_). In contrast, this region is well defined in the final electron density map of PtGSTU20^GSH^ and PtGSTU20^GSPAP^ and adopts the same conformation as observed in known GSTU structures including PtGSTU19^APO/GSH^. This region contains the invariant Lys_39_ that stabilizes the C-terminal carboxylate group of glutathione (Figure 2, Supplementary Figure S5). PtGSTU19 and PtGSTU20 differ by a single amino acid between residues 34 and 51 (Ala_37_ and Thr_37_ in PtGSTU19 and 20, respectively). This region has the same crystalline environment in both structures because the crystals of PtGSTU19 and 20 are isomorphic. The difference could be explained by some additional intramolecular interactions in PtGSTU19. The lateral chain of Glu_33_ in PtGSTU19 (Val_33_ in PtGSTU20) forms two hydrogen bonds that are obviously absent in PtGSTU20. The side chain of Leu_37_ in PtGSTU19 (Leu_37_ in PtGSTU20) is in van der Waals contact with Phe_9_, which is a serine residue in PtGSTU20. In any case, this region (Glu_34_ to Lys_51_) is one of the regions with the highest B factors in PtGSTU19^APO^.

Structural alignment performed with mTM-align (Dong et al., 2018) using the 12 structures of GSTUs available in the RCSB Protein Data Bank (http://www.rcsb.org/, (Burley et al., 2019)), including one from poplar (PtGSTU30, PDB IDs 5J4U and 5J5N) (Yang et al., 2019), suggests GSTU7 from *Salix babylonica* (SbGSTU7, PDB ID 7DWE) and GSTU1 from *R. communis* (RcGSTU1, PDB ID 4J2F) as the closest structural homologs of PtGSTU19 and 20. The similarity trees based on pairwise TM-scores separate the GSTUs into two clades, one of which includes SbGSTU7, RcGSTU1, PtGSTU19 and PtGSTU20 isoforms (Supplementary Figure S8). mTM-align generates an interesting sequence alignment highlighting the common regions, *i.e.*, regions with no gap and with pairwise residue distances less than 4Å (Figure 2). Almost the entire N-terminal domain is a common region, while the C-terminal domain is interspersed with many variable regions located mainly in the loops between the helices. Although these observations have already been made in several studies on GSTs, our alignment also points to variable regions in helices α4, α5, α6, and α9. It is often difficult to explain why several isoforms are grouped because this is the result of an overall effect at the level of primary and three-dimensional structures. In the case of the subclass containing RcGSTU, SbGSTU7, PtGSTU19 and PtGSTU20, specific residues are concentrated in the helices α7 and α8 (Figure 2). This region has no known role in substrate binding or dimerization but could interact with protein partners through protein–protein interactions. Interestingly, this is the case for AtGSTU20 (also known as FIP1), in which the α5–α6 loop and α7 helix interact with the jasmonate amido synthetase FIN219 during regulation of the jasmonate signal (Chen et al., 2017).

Of the crystallographic studies of GSTUs, only three report GSTU structures both in apo form and in complex with glutathione. The first one focuses on GSTU1 from *Mangifera indica* in which GSH binding induces structural changes in three loops. The main change is located in the loop between α5 and α6 helices, which is not part of the G- and H-sites (Valenzuela-Chavira et al., 2017). The second study concerns GSTU23 from *A. thaliana* in which only slight local conformational changes were noted upon GSH binding (Tossounian et al., 2018). The last one presents the structure–function relationships of GSTU6 and 7 isoforms from *S. babylonica* where no difference was reported between apo and glutathione-bound forms (Zhuge et al., 2022). In PtGSTU19 and 20, glutathione binding induces two distinct stabilizations, β2 to β3 region for PtGSTU20 and α6 helix for PtGSTU19. PtGSTU20 has a valine residue at the beginning of the β2 to β3 region and not a glutamic acid as the other known GSTU crystal structures (Figure 2). Indeed, this latter residue participates in the stabilization of the region between β2 and β3 even in the absence of glutathione (see earlier). Structural investigations between apo- and GSH-bound forms in other GST classes (alpha, pi, delta, epsilon, and yeast GTT) also revealed different behaviors across classes (Wongsantichon et al., 2012). When a significant structural change is observed, α2 helix most often undergoes the most prominent rearrangement, as in the case of PtGSTU20. The other regions involved surround the H-site as the C-terminus of α4 as well as most of the loop to α5 and the C-terminus of α8 (Wongsantichon et al., 2012). In PtGSTU19, this is α6, which is also part of the H-site. To our knowledge, this is the first time that this helix is observed in two conformations in the apo form and in one conformation in the GSH-bound form. The α6 helix is located in the heart of the GST subunit and was found to be stable due to the presence of an N-capping motif (Rossjohn et al., 2000; Cocco et al., 2001; Allocati et al., 2006). The latter contains a quasi-invariant aspartate residue in GSTs (Asp_152_ in PtGSTU19 and 20) whose side chain stabilizes the N-terminal side of α6. This study, as well as others, shows that even two closely related GSTs/enzymes may appear to have significantly different local dynamic properties while having close kinetic constants.

### Both PtGSTU19 and 20 Interact With Flavonoids *In Vitro*

**FIGURE 4:**
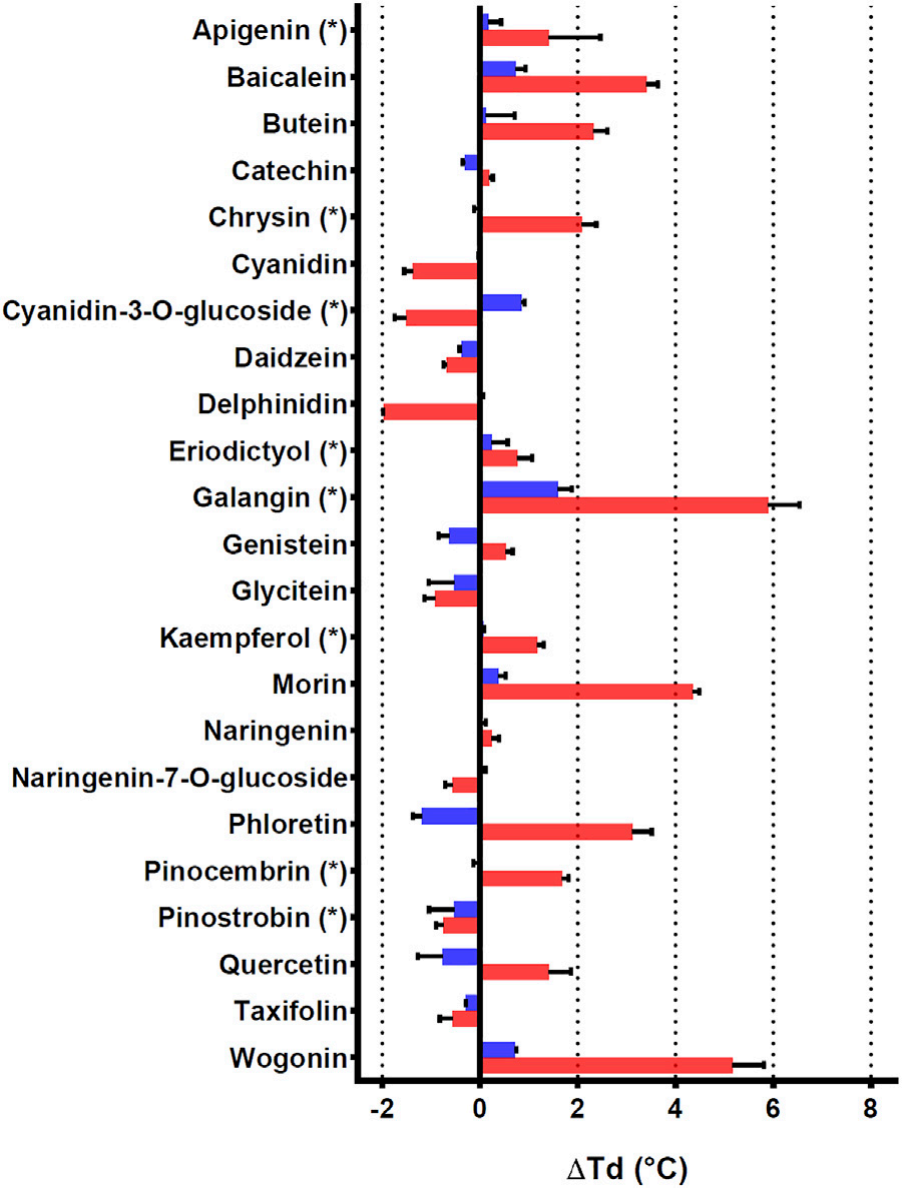
Effects of 23 flavonoids on the thermostability of PtGSTU19 and 20 isoforms. Thermostability of PtGSTU19 (blue bars) and 20 (red bars) isoforms has been analyzed by using 20 μM of protein with or without 100 µM of chemical compounds diluted in 8% DMSO (Supplementary Table S3). The denaturation temperature difference (ΔTd) corresponds to the difference between the denaturation temperature of the protein in the presence of a potential ligand and a reference assay in which the potential ligand is replaced by an equivalent DMSO concentration.

To further characterize PtGSTU19 and 20 at the biochemical level, we sought to identify their potential ligands. Interaction of the apo form of PtGSTU19 and 20 with a set of different classes of chemical compounds including coumarins, flavonoids, terpenes, peroxides, and GST substrates have been screened using thermal shift assay (TSA) (Supplementary Table S2). In these experiments, the thermal denaturation of the proteins is followed by monitoring the fluorescence enhancement of a probe (SYPRO Orange) that binds to protein hydrophobic patches upon denaturation process in the presence or absence of chemical compounds. This rapid and simple method, which can also be used to screen buffer conditions, ligands, cofactors, and drugs, has been successfully used to detect interactions between fungal GSTs and libraries of molecules (Perrot et al., 2018; Schwartz et al., 2018). A few compounds significantly increased the stability (variation of the denaturation temperature ∆Td > 5°C, ∆Td being the difference in melting temperature of the protein incubated in the absence and presence of the molecule) of both recombinant proteins, often with a more pronounced effect for PtGSTU20 (Supplementary Figure S9). Surprisingly, we observed little or no change in the denaturation temperature in the presence of GSH (or GSSG), which is known to have a stabilizing effect on GSTs. GS-PAP (+8.39°C and +9.62°C with PtGST19 and 20, respectively) had a much stronger stabilizing effect on PtGSTU19 and 20, which can be explained by the interaction/recognition of both GS and acetophenone moieties by the proteins. Conversely, a few chemical compounds usually had a destabilizing effect on both proteins. If we focus only on the molecules that stabilized PtGSTU19 and 20 the most (*i.e.,* ∆Td > 4°C), we found mainly molecules from the flavonoid family such as baicalein, morin, and quercetin. These findings prompted us to test a larger set of flavonoids, including some of the flavonoids documented in poplar (apigenin, chrysin, cyanidin-3-*O*-glucoside, eriodictyol, galangin, kaempferol, pinocembrin, and pinostrobin) (Supplementary Table S3 and Figure 4). Again, the stabilizing effect of these molecules (apigenin (+1.41°C), baicalein (+3.40°C), butein (+2.32°C), chrysin (+2.07°C), galangin (+7.42°C), morin (+4.36°C), phloretin (+3.11°C), pinocembrin (+1.68°C), and wogonin (+5.17°C) notably) was more marked on PtGSTU20 (Figure 4), suggesting that the latter has a better affinity for these compounds or that the protein adopts a conformation more prone to bind ligands. These findings were confirmed by determining the inhibitory constant of some of these molecules for which values were measurable (galangin, morin, baicalein, and pinocembrin) using a GSH-conjugating assay with PITC as substrate (Table 3). These flavonoids exhibit a stronger inhibitory effect on PtGSTU20 (*K*_i_ of the order of a hundred or even 10 µM) and thus a stronger interaction with this protein.

**TABLE 3:** Denaturation temperatures and inhibition constants (*K*_i_) of PtGSTU19 and PtGSTU20 activity by flavonoids and GS-PAP.

	PtGSTU19		PtGSTU20	
	*K*_i_ (µM)	ΔTd (°C)	*K*_i_ (µM)	ΔTd (°C)
**Baicalein**	35.2 ± 2.8	0.73 ± 0.20	7.1 ± 0.6	3.40 ± 0.24
Galangin (*)	156.8 ± 28.7	1.59 ± 0.29	43.8 ± 0.6	5.89 ± 0.64
Chrysin (*)	ND	−0.05 ± 0.07	ND	2.07 ± 0.31
**Morin**	72.9 ± 5.3	0.37 ± 0.16	16.0 ± 0.9	4.36 ± 0.12
Pinocembrin (*)	152.1 ± 13.5	−0.01 ± 0.12	61.2 ± 10.5	1.68 ± 0.13
**GS-PAP**	2.3 ± 0.8	4.99 ± 0.28	5.1 ± 2.1	6.48 ± 0.17

Inhibition constants have been determined toward GSH-conjugating reactions using PITC as a substrate. **∆**Td corresponds to modification of the denaturation temperature which is significantly different when the protein is incubated with only DMSO. ND means that no inhibition was detected. *K*_i_ was determined with GraphPad Prism 8 software using the mixed model inhibition. Flavonoids found in poplar are highlighted by an asterisk (*).

We attempted to co-crystallize PtGSTU19 and 20 with different flavonoids. Although we obtained colored crystals for PtGSTU19 and 20 in most cases, the electron density maps showed residual peaks only in the active site of PtGSTU20. Complex structures of PtGSTU20 were solved with two flavonols (galangin, U20^GAL^; morin, U20^MOR^), one flavone (baicalein, U20^BAI^), and one flavanone (pinocembrin, U20^PIN^). Among the four complex structures, only the refined morin model was very well defined in the final electron density map (Supplementary Figure S10). The other flavonoids were refined with partial occupancies ranging from 0.7 to 0.8. The ligands were placed in the putative H-site of PtGSTU20 in a similar manner as the phenylacetophenone moiety of the GS-PAP inhibitor (see earlier). The flavonoids were not refined in the same orientation. The bottom of the pocket is occupied by the benzopyrone ring system in the case of the two flavonols, while it is the phenyl ring in the case of baicalein and pinocembrin. The intermolecular interactions will be described only in the case of the U20^MOR^ complex. The phenyl ring is surrounded by residues from the β1–α1 loop (Val_11_ and Ser_12_), the α1 helix (Pro_13_ and Phe_14_), and the α9 helix (His_208_). His_208_ is hydrogen bonded to the 4′-hydroxyl group of morin. The benzopyrone moiety sits in a mainly aliphatic pocket well delineated by residues from helices α4 (Thr_104_, Phe_107_), α6 (Leu_157_, Phe_158_, Trp_161_, Leu_162_, and Ile_165_), and α9 (Met_209_) (Figure 3). The 7-hydroxyl group is hydrogen bonded to the carbonyl group of Leu_157_. Most of the aliphatic residues are conserved in PtGSTU19. Its α6 helix has two conformations (see earlier): one is similar to that found in PtGSTU20 and the second significantly reduces the volume of the aliphatic pocket. The disorder of this α6 helix could explain why it was not possible to obtain crystallographic structures of PtGSTU19 in complex with flavonoids.

In addition to their catalytic properties, numerous plant GSTs also participate in the binding and transport of a wide range of small heterocyclic ligands such as flavonoids, including anthocyanins, and polyphenols through noncatalytic, ligandin properties (Sylvestre-Gonon et al., 2019). The so-called “ligandin” sites, also called L-sites, are used for the binding of xenobiotic molecules without a catalytic mechanism (Habig et al., 1974; Mannervik and Danielson, 1988). In this case, the supposed role of the GSTs is to intracellularly sequester the toxic molecule and/or to transport it to another detoxification site (Hayes et al., 2005). Depending on the class of GST and the nature of the molecule, different L-sites have been identified by crystallography. The first GST structure complexed with a ligand that binds elsewhere than the active site is the GST Mu from *Schistosoma japonica* in complex with the anthelmintic praziquantel (McTigue et al., 1995). This molecule binds at the interface of the GST dimer near the α3 and α4 helices of each monomer. A ligandin site of similar localization to GST Mu was identified from the structure of mutated human GST Omega in complex with the substrate GS-nitroacetophenone (Brock et al., 2013). This substrate does not bind to the active site of GSTO but near the dimeric interface along the α3 and α4 helices. In the case of human GST Pi, a ligandin site similar to the H-site has been reported. This site binds large polyaromatic molecules, such as sulfasalazine or cibacron blue. Despite the binding of these molecules in the vicinity of the G-site, no glutathionylation reaction was detected, suggesting a noncatalytic role (Oakley et al., 1999). In plants, three main L-sites have been described in one GSTU and one GSTF. L1-site has been localized in GSTU4 from *G. max* complexed to (4-nitrophenyl)methanethiol in each subunit of the dimer in a hydrophobic surface pocket defined by residues from helix α1, strand β2, and helix α8 (Axarli et al., 2016). In turn, L2- and L3-sites have been identified in GSTF2 from *A. thaliana* in complex with two indole derivatives and two flavonoids, respectively, between helices α4 and α7 in each monomer and at the base of the dimer interface involving helices α3 of one subunit and α4 of its neighbor (Ahmad et al., 2017). In the present study, we discovered a fourth L-site for plants that occurs in the H-site of the PtGSTU20. The binding of the flavonoids does not result in enzymatic catalysis in the presence of glutathione, suggesting a role in the metabolism or trafficking of flavonoids as observed for other plant GSTs (Kitamura et al., 2004; Dixon and Edwards, 2010; Sun et al., 2012).

## Conclusion

In this study, we focused on the two paralogous proteins GSTU19 and GSTU20 from *P. trichocarpa*. These two paralogs would have diverged from a common ancestor of *P. trichocarpa* and *P. yatungensis*, from which significant differences emerged in three-dimensional structures. A major difference is in the active site at α6 helix, where the primary structures differ most. This region is considered the bottom of the electrophilic substrate site (H-site) (Sylvestre-Gonon et al., 2019). Two-thirds of the α6 helix is flexible in PtGSTU19 while only one conformation is observed in PtGSTU20. This last conformation, observed for the first time in a GSTU, creates a very deep pocket. The two paralogs PtGSTU19 and 20 showed similar catalytic performances despite this structural difference in the active site. Several explanations are possible: the electrophilic substrates tested are not disturbed by the disorder of the α6 helix in PtGSTU19; the substrates (glutathione and electrophilic substrates) induce a stabilization of the active site as observed in the structure of PtGSTU19 in complex with glutathione. The deep pocket appears to allow binding of polyphenols without catalytic activity. Studies in solution and in the crystal show that PtGSTU20 is the isoform most capable of binding the tested molecules. This difference between the two paralogs can be seen as an emerging evolution toward new functions such as the transport of specialized metabolites.

## Data Availability

The datasets presented in this study can be found in online repositories. The names of the repository/repositories and accession number(s) (7ZS3, 7ZVP, 7ZZN, 8A08, 8A0I, 8A0O, 8A0P, 8A0Q and 8A0R) can be found at: http://www.wwpdb.org/.
