# Supplementary material for: Biochemical and Structural Insights on the Poplar Tau Glutathione Transferase GSTU19 and 20 Paralogs Binding Flavonoids
Source: Front Mol Biosci. 2022 Aug 12;9:958586. doi: 10.3389/fmolb.2022.958586 (PMC9412104; doi:10.3389/fmolb.2022.958586)
Supplement: Supplementary file 1 [file DataSheet1.pdf]

Biochemical and structural insights on the poplar Tau glutathione transferase GSTU19 and 20 paralogs binding flavonoids

Elodie Sylvestre-Gonon[1], Laura Morette[1,2], Morgane Viloria[2], Sandrine Mathiot[2], Alexis Boutilliat[1], Frédérique Favier[2], Nicolas Rouhier[1], Claude Didierjean[2,*] and Arnaud Hecker[1,*]

Supporting information

Table of contents

Table S1. Oligonucleotides used in the study.......... 2

Table S2. List of metabolites used for thermal shift assays. 3

Table S3. List of the 23 metabolites used for thermal shift assays. 4

Table S4. Kinetic parameters of PtGSTU19 and 20 variants toward model substrates. 5

Figure S1. Genetic map displaying GST genes over *Populus trichocarpa* chromosomes.......... 6

Figure S2 Phylogenetic tree of poplar GSTUs.......... 7

Figure S3. Purification of PtGSTU19 and PtGSTU20 isoforms. 8

Figure S4. Stereoview of the superimposition of PtGSTU19 and 20 ribbons.......... 9

Figure S5. Binding of Glutathione in the G-site of PtGSTU20. 10

Figure S6. Views highlighting the putative H-site of PtGSTU19 and 20. 11

Figure S7. Views highlighting the difference between the apo-form and the GSH-complex of both PtGSTU19 and 20.......... 12

Figure S8. Structure-based similarity tree of Tau GSTs based on the pairwise TM-scores.......... 13

Figure S9. Effects of different compounds from the chemical library on the thermostability of PtGSTU19 and PtGSTU20 isoforms.......... 14

Figure S10. Stereoviews of the 2mFo-DFc maps in the putative H-site of PtGSTU20 in $U20^{MOR}$, $U20^{GAL}$, $U20^{BAI}$ and $U20^{PIN}$ crystal structures.......... 15

References.......... 16

Table S1. Oligonucleotides used in the study.

Name	Sequence
**PtGSTU19 (Potri.008G174900)**	
**PtGSTU19/20-B*sm*BI/*Nco*I-For**	5’-cccccccgtctcccatggcagatgtgaagctgcat
**PtGSTU19/20-B*am*HI-Rev**	5’-ccccggatccctacttgttcatcattttc-3
**PtGSTU20 (Potri.008G175000)**	
**PtGSTU19/20-N*co*I-For**	5’-cccccccgtctcccatggcagatgtgaagctgcat
**PtGSTU19/20-B*am*HI-Rev**	5’-ccccggatccctacttgttcatcattttc-3’
**PtGSTU19Y160A**	
**PtGSTU19 Y160A For**	5'-tggctgcaagccaagcaacgaatagtccatacgagatgtcaa-3'
**PtGSTU19 Y160A Rev**	5'-ttgacatctcgtatggactattcgttgcttggcttgcagcca-3'
**PtGSTU19Y160C**	
**PtGSTU19 Y160C For**	5'-ggctgcaagccaacaaacgaatagtccatacgagatgt-3'
**PtGSTU19 Y160C Rev**	5'-acatctcgtatggactattcgtttgttggcttgcagcc-3'
**PtGSTU19Y160F**	
**PtGSTU19 Y160F For**	5'-ggctgcaagccaaaaaacgaatagtccatacgagatgt-3'
**PtGSTU19 Y160F Rev**	5'-acatctcgtatggactattcgttttttggcttgcagcc-3'
**PtGSTU20C160Y**	
**PtGSTU20 C160Y For**	5'- ggcttcaagccaataagtgaagagtccaaatgagatgt-3'
**PtGSTU20 C160Y Rev**	5'- acatctcatttggactcttcacttattggcttgaagcc-3'

Table S2. List of metabolites used for thermal shift assays.

Metabolites	Metabolites	Metabolites
(−)-Epicatechin	4-amino-naphtalen-1-ol	Menadione
1,4 benzoquinone	4-aminotoluene-3-sulfonic acid	Morine
1-bromo-2,4,6-triisopropylbenzene	4-chloro-3-nitrobenzoic acid	Oxidized glutathione
1-chloro-2,4-dinitrobenzene	4-nitrocatechol	Phenyl isothiocyanate
2-(4-hydroxyphenylazo)benzoic acid	Acetosyringone	Piceol
2,2',4,4' tetrahydroxybenzophenone	Anthrone	p-xylene-2-sulfonic acid
2,3-dihydroxybenzoic acid	Ascorbic acid	(2R,3R)-Dihydroquercetin
2,4 dihydroxybenzophenone	Baicalein	Resorcinol
2,4-dichlorophenoxyacetic acid	Butylated hydroxytoluene	Sinapic acid
2,4-Pentanedione peroxide 34%	Catechin hydrate	Syringaldehyde
2,6 dichloro 1,4 benzoquinone	Coniferaldehyde	Taxifoline
2-bromo-1,1,3-trimethoxypropane	Cumene hydroperoxide	Tert-butyl Hydroperoxyde
2-bromo-1-tetralone	Cyclobutane malonyl peroxide	Vanillic acid
2-bromo-2,6-dimethoxybiphenyl	Esculetin	Vanillin
2-bromo-2-phenylacetophenone	Fraxetin	GSH (reduced glutathione)
2-ethylhexyl-4-methoxycinnamate	Gallic acid	GSSG (oxidized glutathione)
2-hydroxy-4-methoxybenzophenone	Gallotannin	GS-phenylacetophenone
3-(dimethylamino)benzoic acid	Indole-3-carboxaldehyde	
4,4'-dihydroxybenzophenone	Isopimpinellin	

Table S3. List of the 23 metabolites used for thermal shift assays.

Metabolites	Metabolites	Metabolites
Apigenin (*)	Delphinidin	Naringenin-7-O-glucoside
Baicalein	Eriodictyol (*)	Phloretin
Butein	Galangin (*)	Pinocembrin (*)
Catechin	Genistein	Pinostrobin (*)
Chrysin (*)	Glycitein	Quercetin
Cyanidin	Kaempferol (*)	Taxifolin
Cyanidin-3-O-glucoside (*)	Morin	Wogonin
Daidzein	Naringenin	*** flavonoid found in poplar**

Table S4. Kinetic parameters of PtGSTU19 and 20 variants toward model substrates.

	CDNB	PITC	BITC	PNP-butyrate	CuOOH
***k*$_{cat}$ (s^{-1})**					
**PtGSTU19**	28.8 ± 2.2	3.8 ± 0.1	7.0 ± 0.1	0.040 ± 0.001	0.17 ± 0.01
**PtGSTU19 Y160A**	7.2 ± 0.9	2.3 ± 0.03	6.5 ± 0.2	0.050 ± 0.002	0.33 ± 0.02
**PtGSTU19 Y160C**	14.0 ± 1.2	3.05 ± 0.05	6.7 ± 0.1	0.050 ± 0.002	0.45 ± 0.03
**PtGSTU19 Y160F**	17.3 ± 1.0	3.9 ± 0.07	8.3 ± 0.2	0.050 ± 0.002	0.49 ± 0.01
**PtGSTU20**	0.59 ± 0.03	5.1 ± 0.1	10.4 ± 0.3	0.020 ± 0.001	0.24 ± 0.01
**PtGSTU19 C160Y**	0.59 ± 0.03	2.03 ± 0.08	4.9 ± 0.1	0.020 ± 0.001	0.14 ± 0.01
***K*$_M$ (µM)**					
**PtGSTU19**	3394 ± 502	48.4 ± 2.5	52.7 ± 3.6	443.5 ± 31.1	196.5 ± 31.1
**PtGSTU19 Y160A**	7042 ± 1379	49.2 ± 2.6	30.6 ± 4.5	534.6 ± 68.1	701.3 ± 82.7
**PtGSTU19 Y160C**	4158 ± 620	40.3 ± 3.0	54.1 ± 3.3	518.3 ± 57.6	1265 ± 139.7
**PtGSTU19 Y160F**	3665 ± 390	31.1 ± 2.5	36.28 ± 3.3	693.2 ± 63.8	347.1 ± 34.6
**PtGSTU20**	1777 ± 193	56.8 ± 4.1	149.6 ± 17.6	329.2 ± 28.1	98.6 ± 15.4
**PtGSTU19 C160Y**	2247 ± 229	58.3 ± 9.2	71.4 ± 9.6	326.2 ± 67.6	490.5 ± 97.5
***k*$_{cat}$/*K*$_M$ ($10^3 M^{-1}.s^{-1}$)**					
**PtGSTU19**	8.9 ± 0.8	79.0 ± 1.1	132.2 ± 0.2	0.100 ± 0.002	0.85 ± 0.04
**PtGSTU19 Y160A**	1.02 ± 0.13	46.8 ± 0.6	211.9 ± 6.2	0.090 ± 0.004	0.48 ± 0.02
**PtGSTU19 Y160C**	3.37 ± 0.29	75.6 ± 1.4	124.2 ± 1.7	0.100 ± 0.003	0.36 ± 0.02
**PtGSTU19 Y160F**	4.68 ± 0.27	125.7 ± 2.1	229.4 ± 4.5	0.080 ± 0.003	1.41 ± 0.04
**PtGSTU20**	0.33 ± 0.01	88.9 ± 1.8	69.8 ± 2.2	0.050 ± 0.001	2.4 ± 0.1
**PtGSTU19 C160Y**	0.26 ± 0.01	34.9 ± 1.4	68.8 ± 1.8	0.060 ± 0.003	0.28 ± 0.02

The apparent K_M values for all compounds were determined for PtGSTU19 and 20 by varying substrate concentrations at a fixed saturating GSH concentration. The apparent K_M and k_{cat} values were calculated with GraphPad Prism 8 software using the Michaelis–Menten equation as non-linear regression model. Results are means ± S.D. (n = 3).

Figure S1. Genetic map displaying GST genes over *Populus trichocarpa* chromosomes.

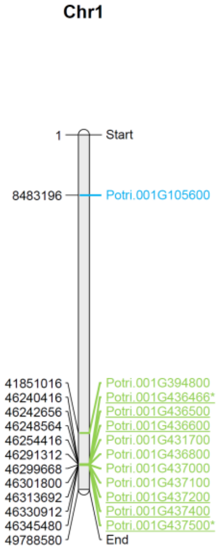

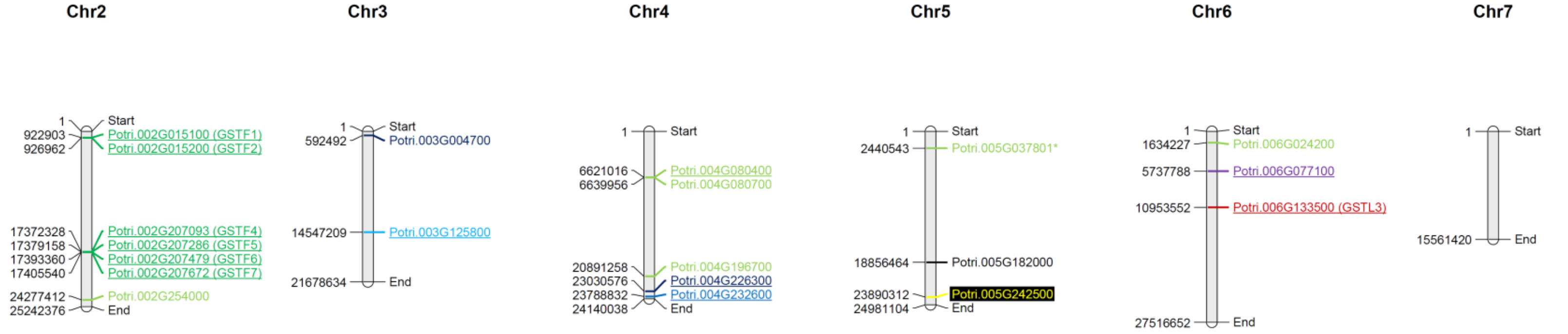

Poplar GST genes have been mapped over the 19 chromosomes using the v4.1 assembly of *Populus trichocarpa* genome available on Phytozome website (version 13). Gene names as well as chromosome numbers (ChrXX) are indicated. Genes on the antisense strand are underlined and probable pseudogenes are indicated by an asterisk (*). This figure has been prepared with MapChart software v2.32 (Voorrips, 2002) according to the following color code: **GSTL** (dark red), **GHR** (red), **DHAR** (orange), **mPGES2** (yellow), **GSTU** (light green), **GSTF** (green), **GSTT** (cyan), **GSTZ** (blue), **EF1Bγ** (dark blue), **MTX** (purple), **TCHQD** (black).

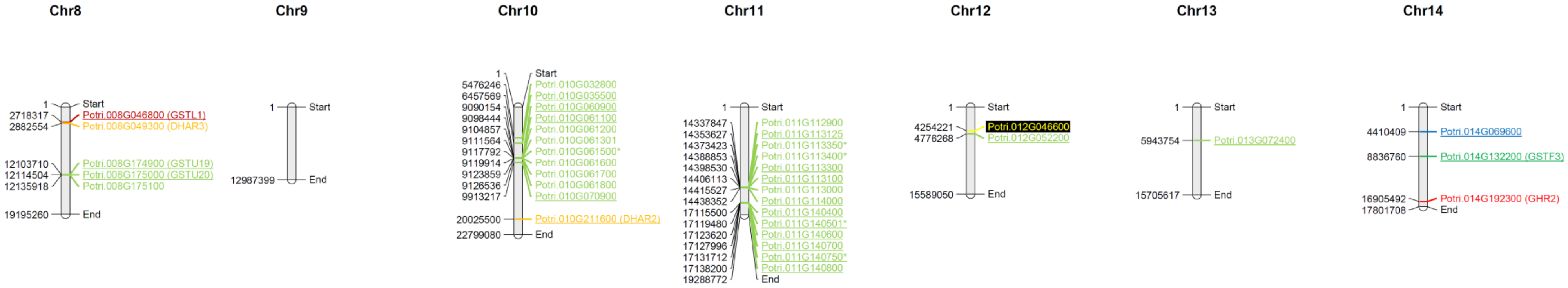

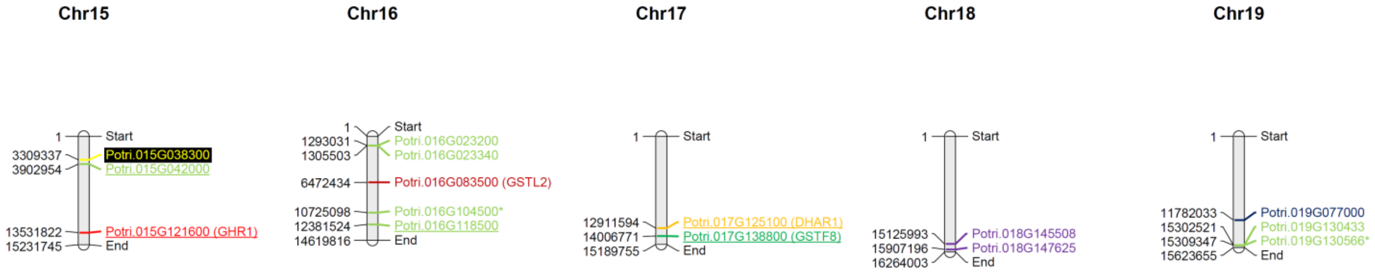

Figure S2 Phylogenetic tree of poplar GSTUs.

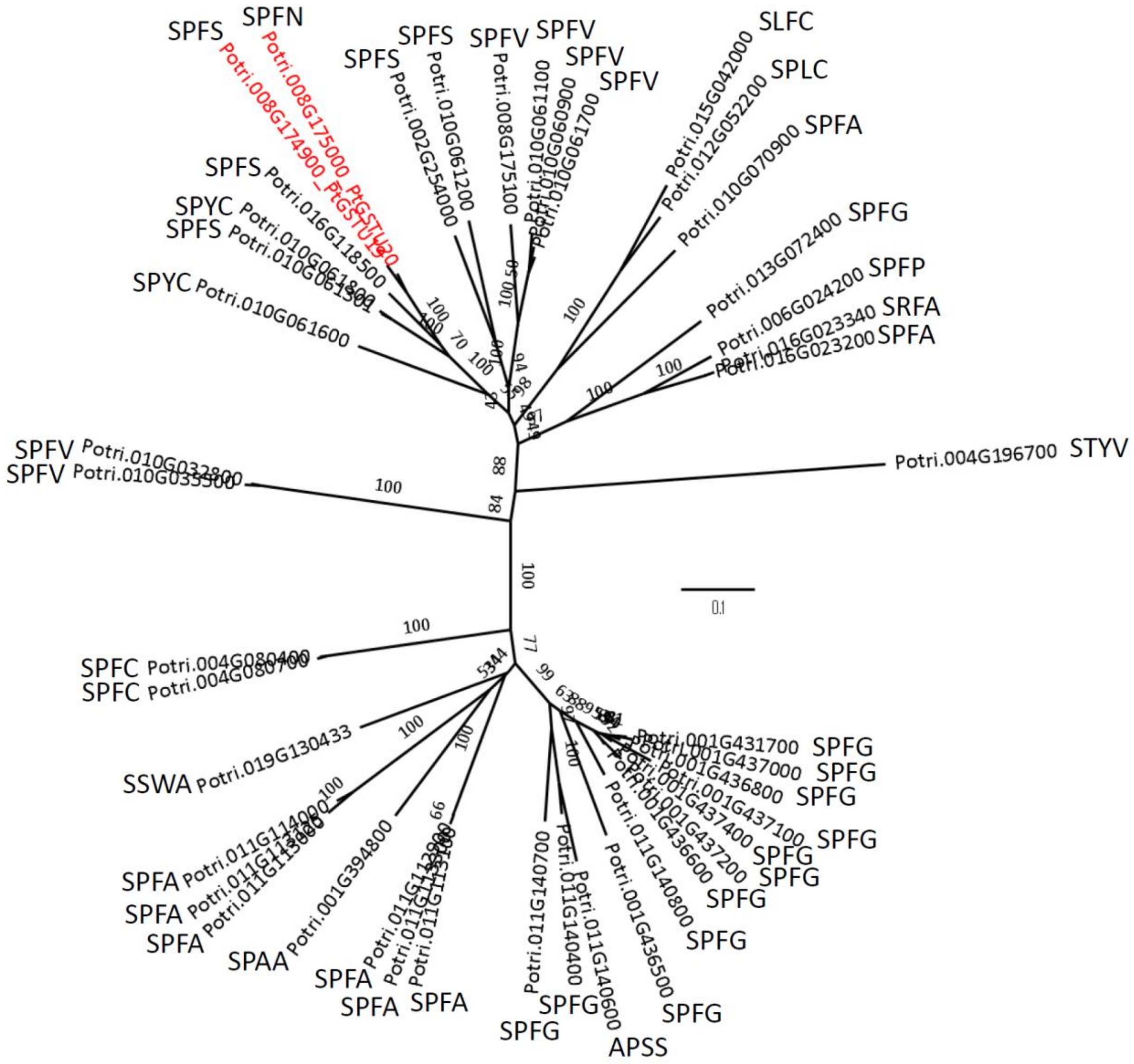

Primary sequences of poplar GSTUs were retrieved from *Populus trichocarpa* genome (v4.1 assembly) and aligned with Clustal Omega implemented in Seaview software (Gouy et al., 2010). The phylogenetic tree was then built using the BioNJ software after curing alignment from hypervariable regions with GBlocks software (Seaview). The robustness of the tree was assessed by the bootstrap method (500 replications). PtGSTU19 and 20 isoforms are highlighted in red and conserved motif in active site is indicated. The scale corresponds to 0.1 substitution per site.

Figure S3. Purification of PtGSTU19 and PtGSTU20 isoforms.

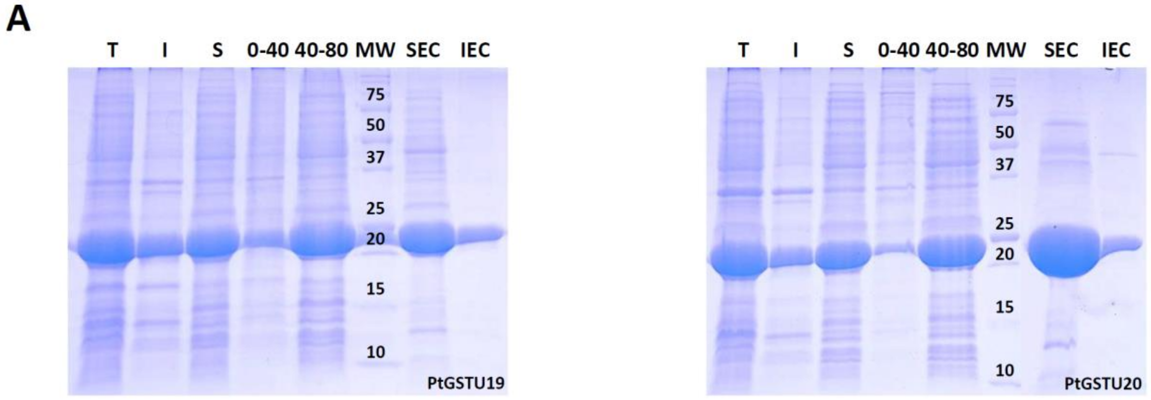

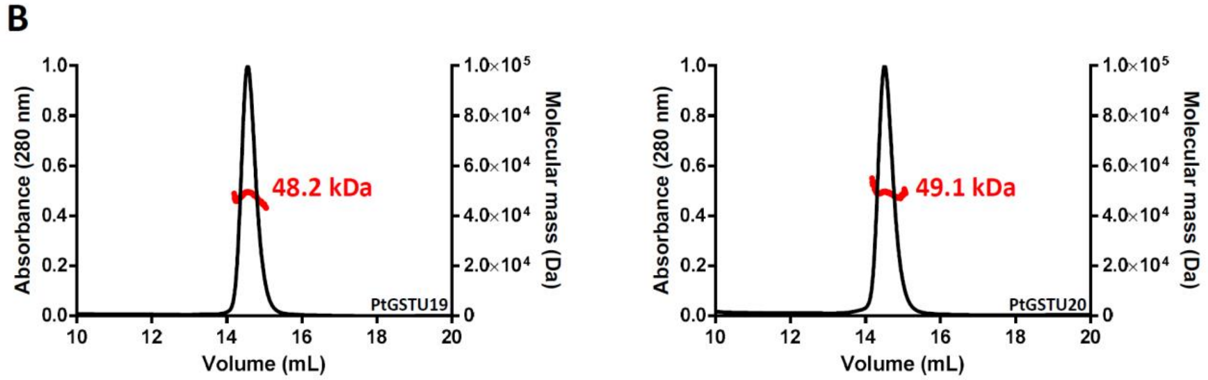

C

Protein	Theoretical molecular mass (Da)	Measured molecular mass (Da)	Δ mass (Da)	Apparent molecular mass (Da)	Retention on GSH-sepharose
**PtGSTU19**	25024,03	24892,56	-131.47	48200	Partially
**PtGSTU20**	24927,01	24796,09	- 130.92	49100	No

A. Coomassie blue-stained sodium dodecylsulfate-polyacrylamide gel electrophoresis (SDS-PAGE) analysis of total (T), soluble (S), insoluble (I) protein fraction from *E. coli* BL21 (DE3) pSBET propagating PtGSTU19 (left) or PtGSTU20 (right)-producing plasmid grown in absence or presence of 0.1 mM IPTG. Fractions collected during ammonium sulfate precipitation step (0%-40% and 40%-80%), size-exclusion chromatography (SEC) and ion-exchange chromatography (IEC) were also presented. MM: molecular mass marker.

B. Purified PtGSTU19 (left) and PtGSTU20 (right) proteins (300 µg in 300 µl of lysis buffer) were analyzed by SEC-MALS using an analytical Superdex200 10/300 column connected to a multi-angle light scattering (MALS) detector (miniDAWN TREOS, Wyatt technology) and a refractometer (T-rEX, Wyatt Technology). Data were processed using Astra 7 software (Wyatt Technology).

C. Molecular masses of purified PtGSTU19 and 20 deduced from primary sequences and determined by mass spectrometry using a Bruker microTOF-Q spectrometer (Bruker Daltonics, Bremen, Germany) equipped with an Apollo II electrospray ionization source with an ion funnel and operated in the negative ion mode. Ability to bind GS-sepharose is also indicated.

Figure S4. Stereoview of the superimposition of PtGSTU19 and 20 ribbons.

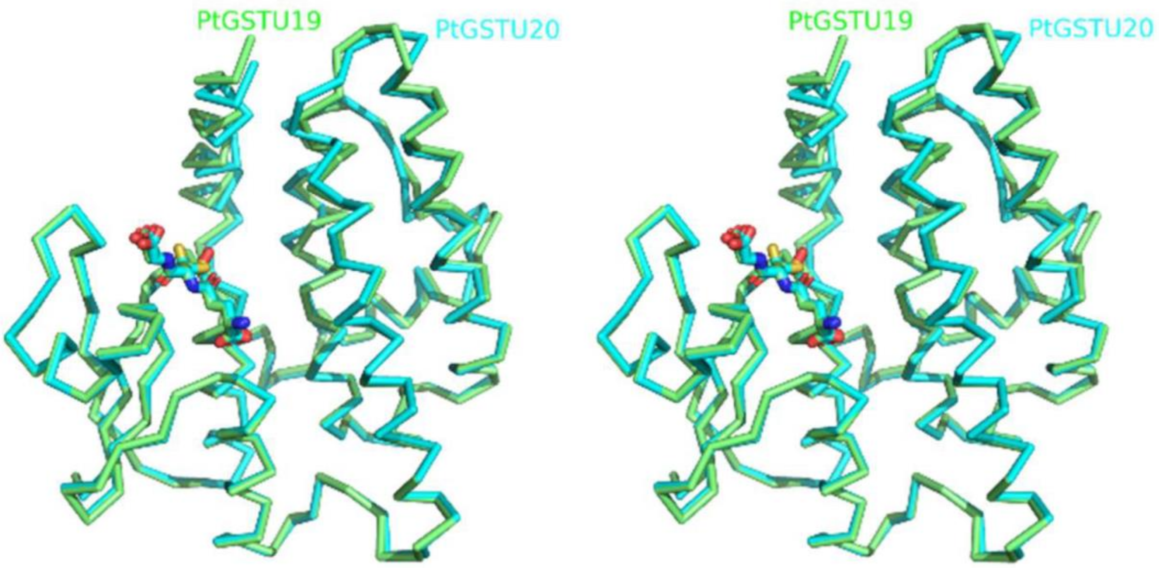

The oxidized glutathione form and both conformations of GSH are shown as sticks in PtGSTU19 and 20, respectively. The carbon atoms are colored green and blue in PtGSTU19 and 20, respectively. All non-carbon atoms are colored according to their types (red, oxygen; blue nitrogen; yellow, sulfur).

Figure S5. Binding of Glutathione in the G-site of PtGSTU20.

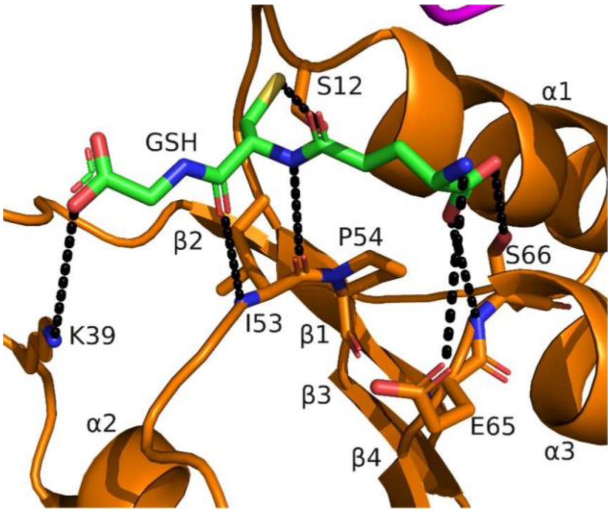

Only one conformation of GSH is shown for clarity. GSH is represented in sticks as the residues surrounding it. Intermolecular contacts are depicted as dashed lines. The N- and C-terminal domains are colored orange and magenta, respectively and GSH is colored green. Non-carbon atoms are colored according to their types (red, oxygen; blue nitrogen; yellow, sulfur).

Figure S6. Views highlighting the putative H-site of PtGSTU19 and 20.

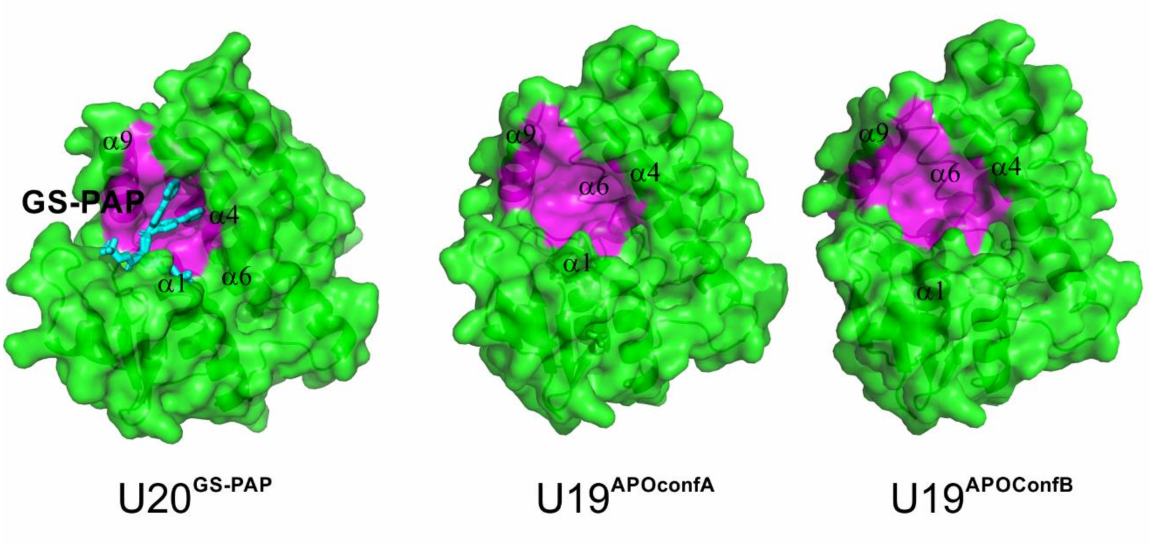

Crystal structures of $U20^{GS-PAP}$ (left) and $U19^{APO}$ ($U19^{APOConfA}$ middle and $U19^{APOConfB}$ right) are shown as transparent surfaces and cartoon representations. GS-PAP (left) is shown as sticks. In $U19^{APO}$, nearly two thirds of the helix $\alpha 6$ was found in two conformations (ConfA (left) and ConfB (right)). Only ConfA was observed in PtGSTU20 structures. H-site is deeper in ConfA than it is in ConfB. The surfaces of the H-sites are highlighted in magenta.

Figure S7. Views highlighting the difference between the apo-form and the GSH-complex of both PtGSTU19 and 20.

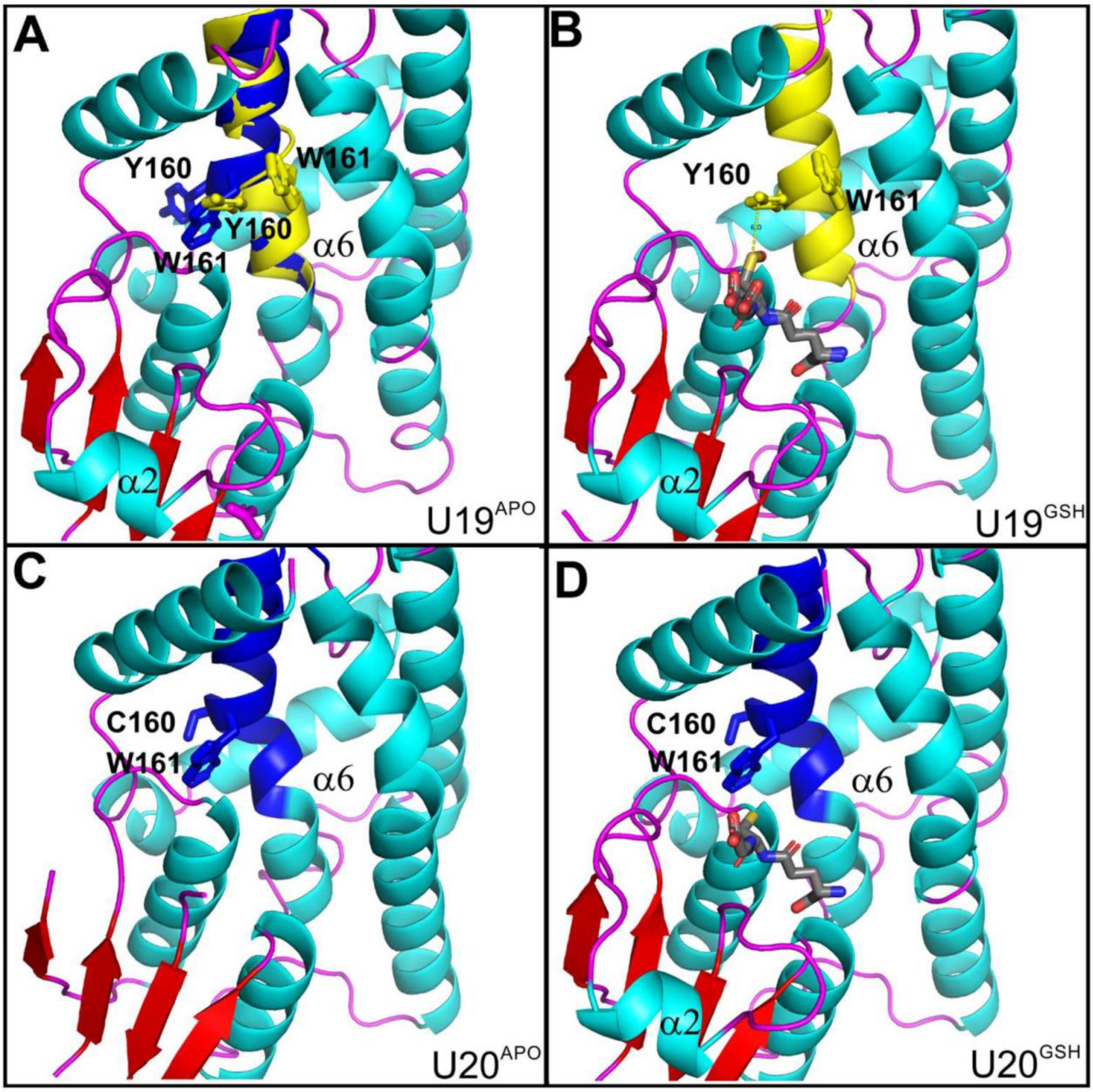

A. Apo form of PtGSTU19. Nearly two thirds of the helix α6 was found in two conformations (ConfA in blue and ConfB in yellow). Tyr160 and W161 residues were in quite distinct orientations in both conformations.

B. PtGSTU19 in complex with GSOH. Only the ConfB was observed. The side chain of Tyr160 points towards the glutathione molecule.

C. Apo form of PtGSTU20. Only ConfA was observed. Cys160 replaces Tyr160 of PtGSTU19. In the apo form, region 34 to 51 (helix α2 and its upstream and downstream loops) was not visible in the electron density maps.

D. PtGSTU20 in complex with GSH. The region 34 to 51 (helix α2 and its upstream and downstream loops) was visible in the electron density maps and adopts the same conformation as observed in PtGSTU19.

Figure S8. Structure-based similarity tree of Tau GSTs based on the pairwise TM-scores.

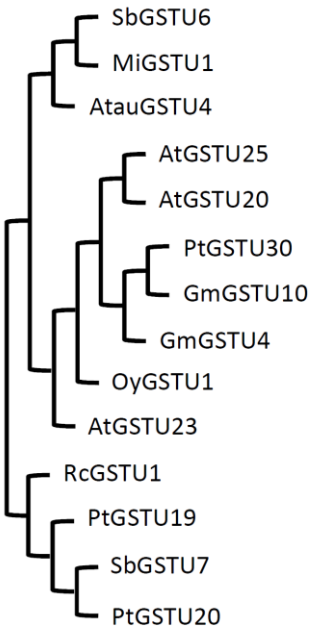

The GSTU crystal-structures were aligned using mTM-align (Dong et al., 2018, see reference in the main text), which generated all pairwise structure alignments, TM-scores for the input set of structures and structure-based similarity tree. The tree includes the following GSTUs: SbGSTU6 (GSTU6 from *Salix babylonica*, PDB ID 7DW2), MiGSTU1 (GSTU1 from *Mangifera indica*, 5G5E), TaGSTU4 (GSTU4 from *Aegilops tauschii*, 1GWC), AtGSTU25 (GSTU25 from *Arabidopsis thaliana*, 5G5A), AtGSTU20 (GSTU20 from *A. thaliana*, 5ECS), PtGSTU30 (GSTU30 from *P. trichocarpa*, 5J4U), GmGSTU10 (GSTU10 from *Glycine max*, 4CHS), GmGSTU4 (GSTU4 from *Glycine max*, 2VO4), OyGSTU1 (GSTU1 from *Oryza sativa*, 1OYJ), AtGSTU23 (GSTU23 from *A. thaliana*, 6EP7), RcGSTU1 (GSTU1 from *Ricinus communis*, 4J2F), PtGSTU19 (this study), SbGSTU6 (GSTU7 from *S. babylonica*, PDB ID 7DWD), PtGSTU20 (this study).

Figure S9. Effects of different compounds from the chemical library on the thermostability of PtGSTU19 and PtGSTU20 isoforms.

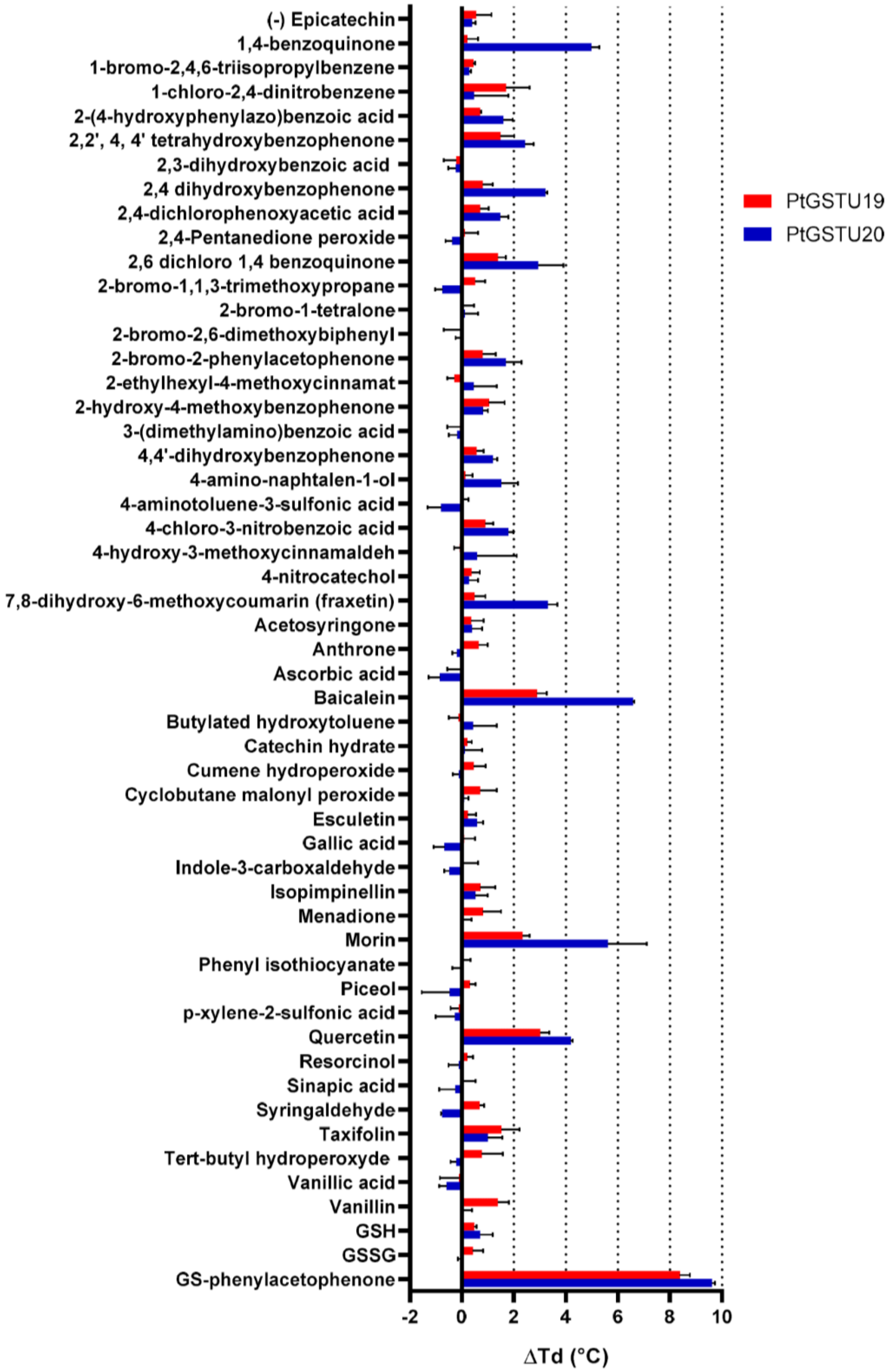

Thermostability of PtGSTU19 (blue bars) and 20 (red bars) isoforms has been analyzed by using 20 µM of protein with or without 100 µM of chemical compounds diluted in 8% DMSO (Table S2). The denaturation temperature difference (ΔTd) corresponds to the difference between the denaturation temperature of the protein in presence of a potential ligand and a reference assay in which the potential ligand is replaced by the equivalent DMSO concentration.

Figure S10. Stereoviews of the 2mFo-DFc maps in the putative H-site of PtGSTU20 in $\mathrm{U20}^{\mathrm{MOR}}$, $\mathrm{U20}^{\mathrm{GAL}}$, $\mathrm{U20}^{\mathrm{BAI}}$ and $\mathrm{U20}^{\mathrm{PIN}}$ crystal structures.

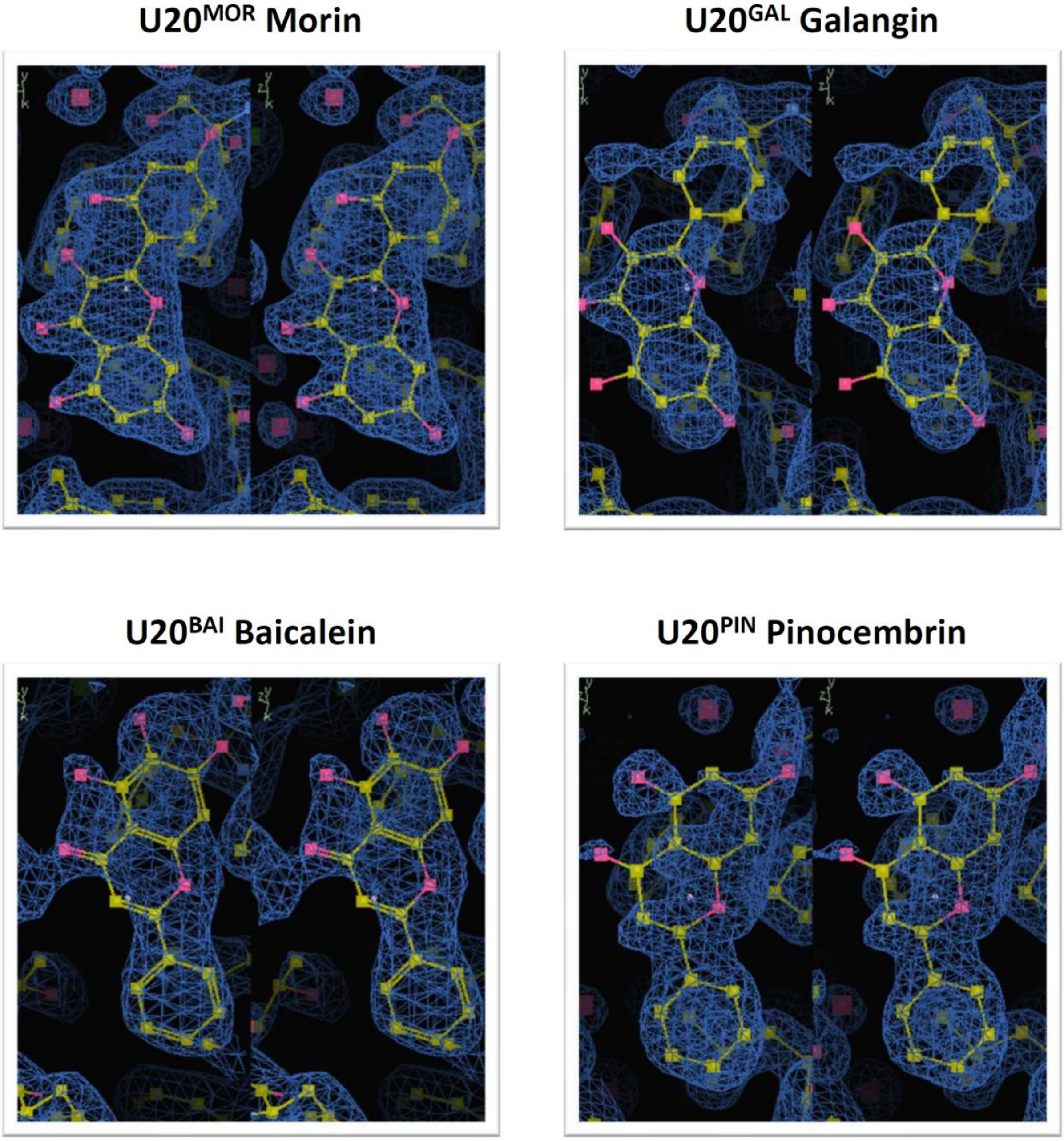

The 2mFo-DFc maps were calculated using Buster (Smart et al., 2012) and the figures were generated by Coot (Emsley and Cowtan, 2004. Each map was contoured at 1.0 σ level where σ is the standard deviation of the map.

References

Dong, R., Pan, S., Peng, Z., Zhang, Y., and Yang, J. (2018). mTM-align: a server for fast protein structure database search and multiple protein structure alignment. *Nucleic Acids Research*. 46(W1), W380-W386. doi: 10.1093/nar/gky430.

Emsley, P., and Cowtan, K. (2004). Coot : model-building tools for molecular graphics. *Acta Crystallogr D Biol Crystallogr* 60, 2126–2132. doi: 10.1107/S0907444904019158.

Gouy, M., Guindon, S., and Gascuel, O. (2010) SeaView Version 4: A Multiplatform Graphical User Interface for Sequence Alignment and Phylogenetic Tree Building. *Molecular Biology and Evolution* 27, 221–224. doi:10.1093/molbev/msp259.

Smart, O. S., Womack, T. O., Flensburg, C., Keller, P., Paciorek, W., Sharff, A., et al. (2012). Exploiting structure similarity in refinement: automated NCS and target-structure restraints in BUSTER. *Acta Crystallogr D Biol Crystallogr* 68, 368–380. doi: 10.1107/S0907444911056058.

Voorrips, R.E. (2002). MapChart: Software for the Graphical Presentation of Linkage Maps and QTLs. *Journal of Heredity* 93, 77-78. doi: 10.1093/jhered/93.1.77.
